# Diabetes mellitus and idiopathic pulmonary fibrosis: a Mendelian randomization study

**DOI:** 10.1186/s12890-024-02961-7

**Published:** 2024-03-20

**Authors:** Quou Kang, Jing Ren, Jinpeng Cong, Wencheng Yu

**Affiliations:** 1 Department of Pulmonary and Critical Care Medicine, The affiliated hospital of Qingdao University, Qingdao University, Qingdao, China; 2 Medical Department of Qingdao University, Qingdao, China

**Keywords:** Idiopathic pulmonary fibrosis, Diabetes mellitus, Type 1 diabetes, Type 2 diabetes, Mendelian randomization, Hyperglycemia

## Abstract

**Background:**

The question as to whether or not diabetes mellitus increases the risk of idiopathic pulmonary fibrosis (IPF) remains controversial. This study aimed to investigate the causal association between type 1 diabetes (T1D), type 2 diabetes (T2D), and IPF using Mendelian randomization (MR) analysis.

**Methods:**

We used two-sample univariate and multivariate MR (MVMR) analyses to investigate the causal relationship between T1D or T2D and IPF. We obtained genome-wide association study (GWAS) data for T1D and T2D from the European Bioinformatics Institute, comprising 29,652 T1D samples and 101,101 T1D single nucleotide polymorphisms (SNPs) and 655,666 T2D samples and 5,030,727 T2D SNPs. We also used IPF GWAS data from the FinnGen Biobank comprising 198,014 IPF samples and 16,380,413 IPF SNPs. All cases and controls in these datasets were derived exclusively from European populations. In the univariate MR analysis, we employed inverse variance-weighted (IVW), weighted median (WM), and MR-Egger regression methods. For the MVMR analysis, we used the multivariate IVW method primarily, and supplemented it with multivariate MR-Egger and multivariate MR- least absolute shrinkage and selection operator methods. Heterogeneity tests were conducted using the MR-IVW and MR-Egger regression methods, whereas pleiotropic effects were assessed using the MR-Egger intercept. The results of MR and sensitivity analyses were visualized using forest, scatter, leave-one-out, and funnel plots.

**Results:**

Univariate MR revealed a significant causal relationship between T1D and IPF (OR = 1.118, 95% CI = 1.021–1.225, *P* = 0.016); however, no significant causal relationship was found between T2D and IPF (OR = 0.911, 95% CI = 0.796–1.043, *P* = 0.178). MVMR analysis further confirmed a causal association between T1D and IPF (OR = 1.133, 95% CI = 1.011–1.270, *P* = 0.032), but no causal relationship between T2D and IPF (OR = 1.009, 95% CI = 0.790–1.288, *P* = 0.950). Sensitivity analysis results validated the stability and reliability of our findings.

**Conclusion:**

Univariate and multivariate analyses demonstrated a causal relationship between T1D and IPF, whereas no evidence was found to support a causal relationship between T2D and IPF. Therefore, in clinical practice, patients with T1D should undergo lung imaging for early detection of IPF.

**Supplementary Information:**

The online version contains supplementary material available at 10.1186/s12890-024-02961-7.

## Introduction

Idiopathic pulmonary fibrosis (IPF) is a chronic fibrotic interstitial pneumonia of unknown etiology characterized by cough, dyspnea, and a progressive decline in lung function that primarily affects the elderly population [1]. The radiological and histological features are predominantly consistent with usual interstitial pneumonia [1]. According to a statistical analysis of 12 countries, the incidence and prevalence rates of IPF range from 0.09–1.30/10,000 and 0.33–4.51/10,000, respectively, exhibiting a sharp increase with advancing age [2]. Globally, the number of patients with IPF is increasing, potentially due to factors such as an aging demographic, heightened disease awareness, and advancements in diagnostic technologies [2]. Patients diagnosed with IPF may have a poorer prognosis than those with many cancers that affect similar populations [3], as evidenced by a median survival rate of only 3–5 years in the absence of treatment [4]. Age, sex, and comorbidities significantly affect the clinical outcomes of patients with IPF, with mortality associated with congestive heart failure (CHF), diabetes mellitus (DM), and cancer, whereas hospitalization is linked to CHF and chronic obstructive pulmonary disease (COPD) [5].

DM is a cluster of systemic metabolic disorders characterized by persistent hyperglycemia, often resulting in chronic damage to the blood vessels, kidneys, retina, and nervous system [6]. DM presents a significant global health challenge, with the number of individuals affected worldwide reaching 424.9 million in 2017 and projected to increase by 48% to 628.6 million by 2045, resulting in substantial economic and social burdens [7].

DM is classified into type 1 diabetes (T1D), type 2 diabetes (T2D), gestational diabetes, and other specific types of diabetes caused by various etiologies [8]. Among these, T1D and T2D account for over 95% of the total diabetic population [8]. Patients with T2D exhibit varying degrees of relative insulin deficiency, which is often accompanied by insulin resistance. In contrast, T1D arises from autoimmune destruction of pancreatic β cells, resulting in an absolute reliance on exogenous insulin for blood glucose regulation [8]. A meta-analysis of observational studies examining the relationship between DM and IPF revealed that individuals with IPF had a 1.54-fold higher likelihood of developing DM than those without IPF; nevertheless, whether DM increases the risk of IPF remains controversial [9]. However, as DM was not classified in this study, causality between the different subtypes of DM and IPF could not be established.

Mendelian randomization (MR) studies have observational designs that employ randomly-assigned genetic variants as phenotypic instrumental variables (IVs) to establish reliable causal inferences regarding exposures and outcomes. Compared to traditional observational studies, MR is less susceptible to confounding or reverse causation [10].

Therefore, in this study, we aimed to examine the causal association between T1D and IPF, and between T2D and IPF using two-sample univariate and multivariate MR (MVMR) methods.

## Methods

### Study design

**Fig. 1:**
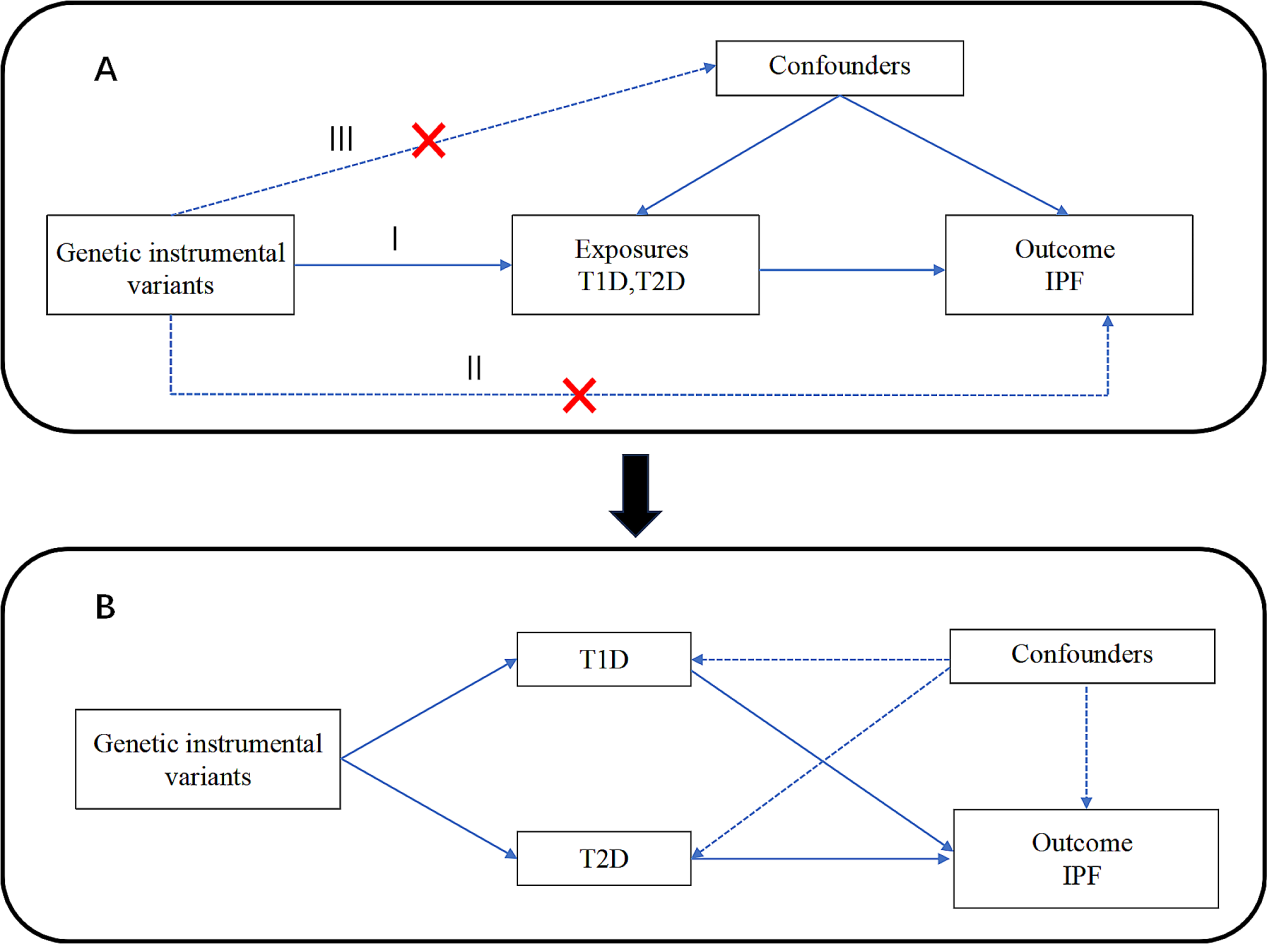
Study design. **(A)** Univariable MR to identify the causal association between T1D or T2D and IPF, which involves IVs and requires three assumptions: (I) strong association between IVs and the exposure of interest; (II) no direct relationship between IVs and outcome, with their effect only indicated through exposure; (III) independence of IVs from any confounding factors affecting both exposure and outcome. **(B)** Multivariable MR to evaluate if T1D remains causal given T2D. MR, Mendelian randomization; T1D, type 1 diabetes; IPF, idiopathic pulmonary fibrosis; T2D, type 2 diabetes; IVs, instrumental variables

The causal relationships between T1D and IPF, as well as between T2D and IPF in the European population, were initially investigated using a two-sample univariate MR analysis. A sensitivity analysis was conducted to ensure data reliability and validity. Subsequently, MVMR analysis was conducted to examine the significance of the causal relationship between diabetes and IPF established by the univariate MR analysis. We applied a set of selection criteria to identify single nucleotide polymorphisms (SNPs) strongly associated with T1D and T2D, which were subsequently used as IVs in the MR analysis: (I) strong association between IVs and the exposure of interest; (II) no direct relationship between IVs and outcome, with their effect only indicated through exposure [11]; and (III) independence of IVs from any confounding factors affecting both exposure and outcome. The current research design is depicted in Fig. 1. This study adhered to the STROBE-MR reporting guidelines (Supplementary Table 4) [12].

### Genome-wide association study (GWAS) data source and instrument selection

We utilized GWAS datasets from various databases for exposure and outcome to mitigate potential bias in causal effect estimation due to sample overlap. The IVs were chosen based on SNPs that were associated with the exposure, as determined by our selection criteria. The T1D and T2D datasets were obtained from the European Bioinformatics Institute. The T1D dataset (ID number: “ebi-a-GCST005536”) comprises 29,652 samples and 101,101 SNPs (https://gwas.mrcieu.ac.uk/datasets/ ebi-a-GCST005536/) [13]. The T2D dataset (ID number: “ebi-a-GCST006867”) comprises 655,666 samples and 5,030,727 SNPs (https://gwas.mrcieu.ac.uk/datasets/ ebi-a-GCST006867/) [14]. The GWAS dataset associated with IPF was established by FinnGen Biobank (ID number: “finn-b-IPF”), and it comprises 198,014 samples and 16,380,413 SNPs (https://gwas.mrcieu.ac.uk/datasets/finn-b-IPF/). The diagnosis of IPF and DM was determined according to the International Classification of Diseases 10th Revision (ICD-10) codes. The detailed characteristics of the exposure and outcome datasets are presented in Table 1.

**Table 1:** Characteristics of exposures and outcome

Variable	GWAS ID	PubMed ID	Cases	Controls	Sample size	Population
**Exposure**						
T1D	ebi-a-GCST005536	25,751,624	6,683	12,173	29,652	European
T2D	ebi-a-GCST006867	30,054,458	61,714	1,178	655,666	European
**Outcome**						
IPF	finn-b-IPF		1,028	196,986	198,014	European

T1D, type 1 diabetes; T2D, type 2 diabetes; IPF, idiopathic pulmonary fibrosis

A genome-wide search was conducted for SNPs (*P* < 5 × 10^− 8^) associated with T1D or T2D exposure, which were used as IVs for genetic prediction. Non-independent SNPs based on linkage disequilibrium (r^2^ < 0.001 within a 10,000 kb aggregation window) were excluded using the European population reference. To ensure independence and exclusivity, these SNPs were searched using Phenoscanner (University of Cambridge, Cambridge, UK; http://www.phenoscanner.medschl.cam.ac.uk/) and any SNPs related to the outcome or confounding factors were excluded. Confounding factors included risk factors other than exposure that may contribute to the outcome. The risk factors associated with IPF include smoking, gastroesophageal reflux, obstructive sleep apnea, herpes virus infection, and certain occupational interstitial lung diseases [15]. To ensure a strong relationship between the IVs and exposure, it is necessary to calculate F-statistics. The F-statistic formula used in this study was F = β^2^/se^2^ (β, effect size(exposure); se, standard error (exposure)). An F-statistic greater than 10 indicates a robust association between the IVs and exposure, thereby avoiding weak instrument bias.

### Mendelian randomization analysis

We conducted a two-sample univariate MR analysis using T1D and T2D as exposures and IPF as the outcome. In particular, we extracted 36 and 118 strongly-associated SNPs from T1D and T2D GWAS datasets, respectively. Among the 36 SNPs that were strongly associated with T1D in the IPF dataset, rs3184504 was excluded because of its correlation with smoking status (Supplementary Table 1). Among the 118 SNPs strongly associated with T2D in the IPF dataset, four palindromic SNPs were excluded: rs13234269, rs1758632, rs2058913, and rs6494307. Additionally, rs2867125 was excluded due to its association with smoking (Supplementary Table 2).

After retrieving SNPs that were strongly correlated with T1D and T2D in Phenoscanner (University of Cambridge), we identified SNPs that were closely associated with both types of diabetes. To mitigate the potential bias of mutually-confounding factors in the MR analysis, we conducted an MVMR analysis. Fifty-nine SNPs strongly associated with either T1D or T2D were extracted from the IPF dataset, and the palindromic SNP, rs17411031, was excluded during reconciliation. Finally, in the MVMR analysis, 26 and 34 effective IVs were employed to estimate the causal effects of T1D and T2D, respectively, on IPF.

In the univariate MR analysis, we employed inverse variance-weighted (IVW), weighted median (WM), and MR-Egger regression methods to examine the causal association between diabetes and IPF. The IVW method was used as the primary analytical approach. To ensure the reliability of our research findings, we employed MR-IVW and MR-Egger regression methods to assess heterogeneity and quantified the results using Cochran’s Q statistics. We employed the MR-Egger intercept test to examine the presence of horizontal pleiotropy while utilizing a scatter plot of effective IVs for estimation of the causal effect of T1D and T2D on IPF to visually assess whether the outcome effect is zero when the IV effect is zero. A non-zero cutoff indicated the existence of horizontal pleiotropy. In addition, to assess the robustness of the findings, a leave-one-out approach was employed to remove SNPs individually and visualize the results using a leave-one-out plot in the MR analysis. The primary method employed for MVMR analysis was the extended multivariate IVW approach, complemented by secondary methods, such as multivariate MR-Egger and multivariate MR-least absolute shrinkage and selection operator (LASSO). The extended IVW and MR-Egger methods were employed to assess heterogeneity, while the MR-Egger intercept was used to evaluate pleiotropic effects. The statistical analyses in this study were performed using the “TwoSampleMR” and “MendelianRandomization” packages in R v4.3.0 (R Foundation for Statistical Computing, Vienna, Austria).

### Ethics

This study relied solely on published GWAS datasets, and all original studies obtained appropriate ethical approval; therefore, separate ethical approval was not required for this study.

## Results

### Univariable MR estimates

**Fig. 2:**
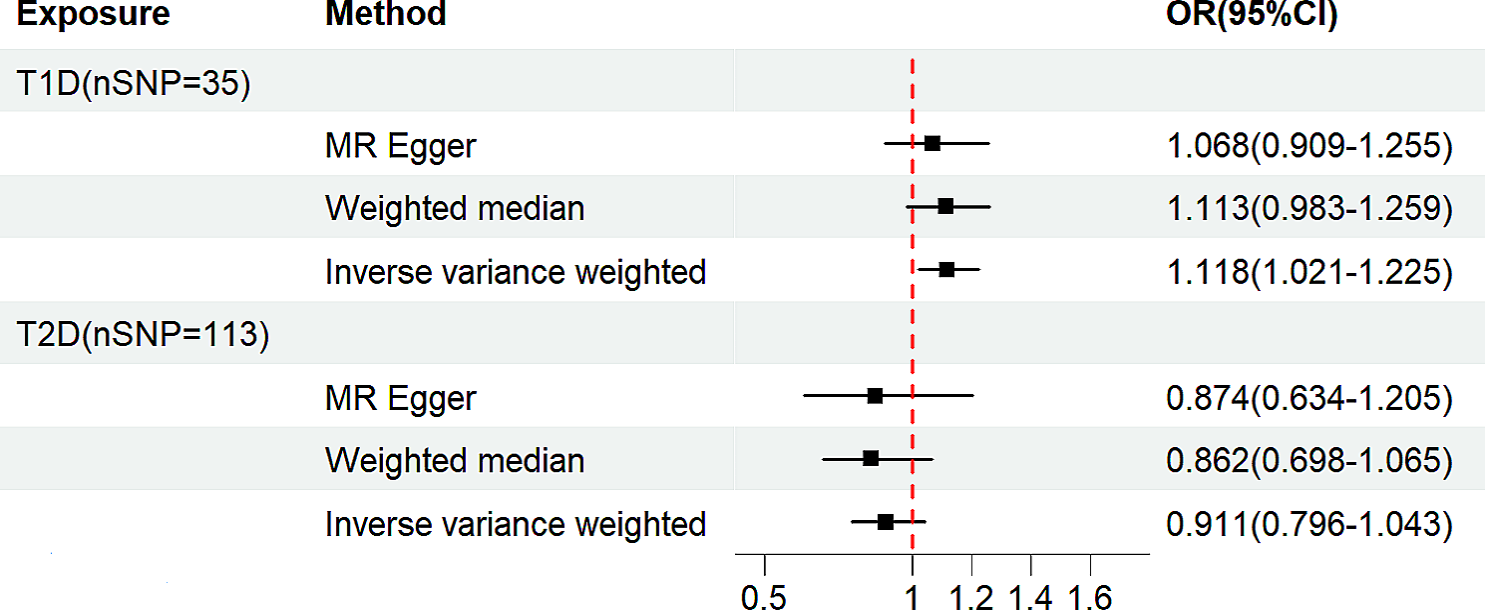
Forest plots of causal effect estimates in univariable Mendelian analysis. T1D, type 1 diabetes; T2D, type 2 diabetes

In the two-sample univariate MR analysis, we included 35 and 113 SNPs associated with T1D and T2D, respectively, to estimate their effects on IPF (Supplementary Tables 1 and 2). The F-statistics of the IVs exceeded 10, indicating their robustness and absence of instrumental variable bias [16]. Table 2; Fig. 2 show the results of the analyses of the causal relationship between T1D or T2D and IPF according to the IVW, MR-Egger, and WM methods. The IVW analysis revealed that T1D was associated with an increased risk of IPF (OR = 1.118, 95% CI = 1.021–1.225, *P* = 0.016), with a mean increase of 11.8% of the likelihood of developing IPF. The WM and MR-Egger methods did not yield statistically significant results (WM: OR = 1.113, 95% CI = 0.983–1.259, *P* = 0.091; MR-Egger: OR = 1.068, 95% CI = 0.909–1.255, *P* = 0.427); however, there was a tendency towards a causal effect of T1D on IPF. On the other hand, investigations examining the impact of T2D on IPF consistently indicated an absence of a causal association between T2D and IPF, as evidenced by *P*-values exceeding 0.05 for IVW, WM, and MR-Egger regression analysis (Table 2; Fig. 2).

**Table 2:** Two-sample univariable MR analysis of the relationship between IPF and type 1 or 2 diabetes

Exposure	Outcome	Method	nSNP	OR	95%CI	*P*-Value
T1D	IPF	IVW	35	1.118	(1.021,1.225)	0.016
		Weighted median		1.113	(0.983,1.260)	0.091
		MR-Egger		1.068	(0.909,1.255)	0.427
T2D	IPF	IVW	113	0.911	(0.796,1.043)	0.178
		Weighted median		0.862	(0.698,1.065)	0.169
		MR-Egger		0.874	(0.634,1.205)	0.412

T1D, type 1 diabetes; T2D, type 2 diabetes; IPF, idiopathic pulmonary fibrosis; IVW, inverse variance weighted

### Multivariable MR estimates

**Fig. 3:**
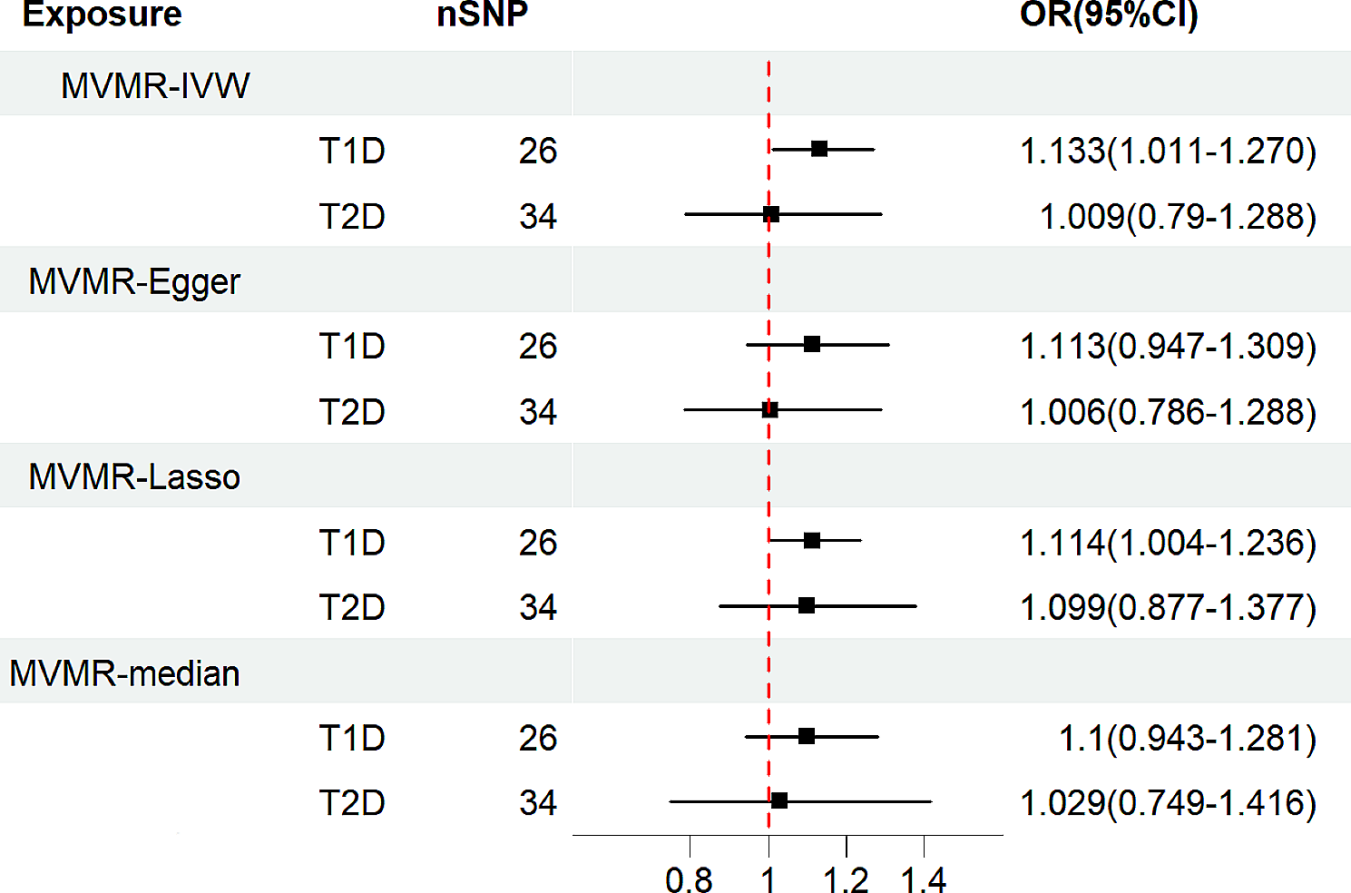
Forest plots of causal effect estimates in multivariable Mendelian analysis. T1D, type 1 diabetes; T2D, type 2 diabetes; MVMR, multivariate Mendelian randomization; IVW, inverse variance weighted

We conducted an MVMR analysis to confirm the persistence of a causal relationship between T1D and IPF even after adjusting for T2D. A total of 58 effective IVs were retained for MVMR analysis, of which 26 SNPs were included in the MR analysis of T1D and IPF and 34 SNPs were included in the MR analysis of T2D and IPF (Supplementary Table 3). When T1D was adjusted for T2D, the results of the multivariate analysis were consistent with the results of the univariate MR, suggesting that T1D was significantly associated with an increased risk of IPF (IVW: OR = 1.133, 95% CI = 1.011–1.270, *P* = 0.032, MR-Lasso: OR = 1.114, 95% CI = 1.004–1.236, *P* = 0.042, Table 3; Fig. 3). The results of the MR-Egger and median methods were not statistically significant, but tended towards a causal effect consistent with that of the IVW and MR-LASSO analyses, indicating that T1D increases the risk of IPF. Additionally, multivariate analysis of the effect of T2D on IPF yielded results consistent with those of the univariate analysis, indicating no causal relationship. Furthermore, all *P*-values from IVW, MR-Egger, MR-LASSO, and median analyses were greater than 0.05 (Table 3; Fig. 3).

**Table 3:** Two-sample multivariable Mendelian randomization analysis of the relationship for T1D or T2D on IPF

Exposure	Outcome	Method	nSNP	OR	95%CI	*P*-value
T1D	IPF	IVW	26	1.133	(1.011,1.270)	0.032
T2D	IPF	IVW	34	1.009	(0.790,1.289)	0.946

T1D, type 1 diabetes; T2D, type 2 diabetes; IPF, idiopathic pulmonary fibrosis

### Sensitivity analysis

**Fig. 4:**
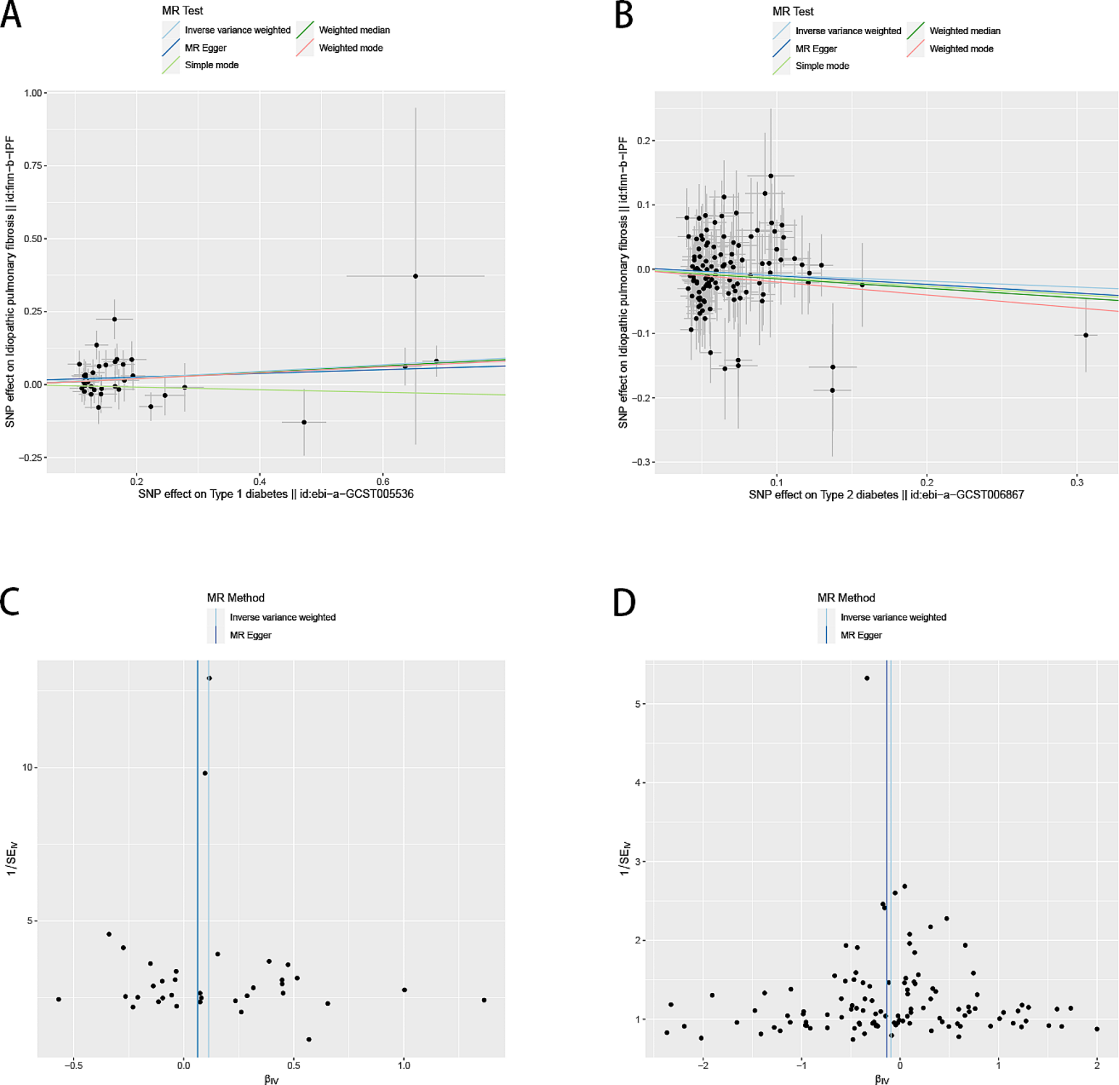
**(A)** Scatter plots of causal effect for T1D on IPF, **(B)** T2D on IPF, **(C)** Funnel plots to visualize overall heterogeneity of MR estimates for the effect of T1D on IPF, **(D)** T2D on IPF.T1D, type 1 diabetes; IPF, idiopathic pulmonary fibrosis; T2D, type 2 diabetes; IVW, inverse-variance weighted; MR, Mendelian randomization

**Fig. 5:**
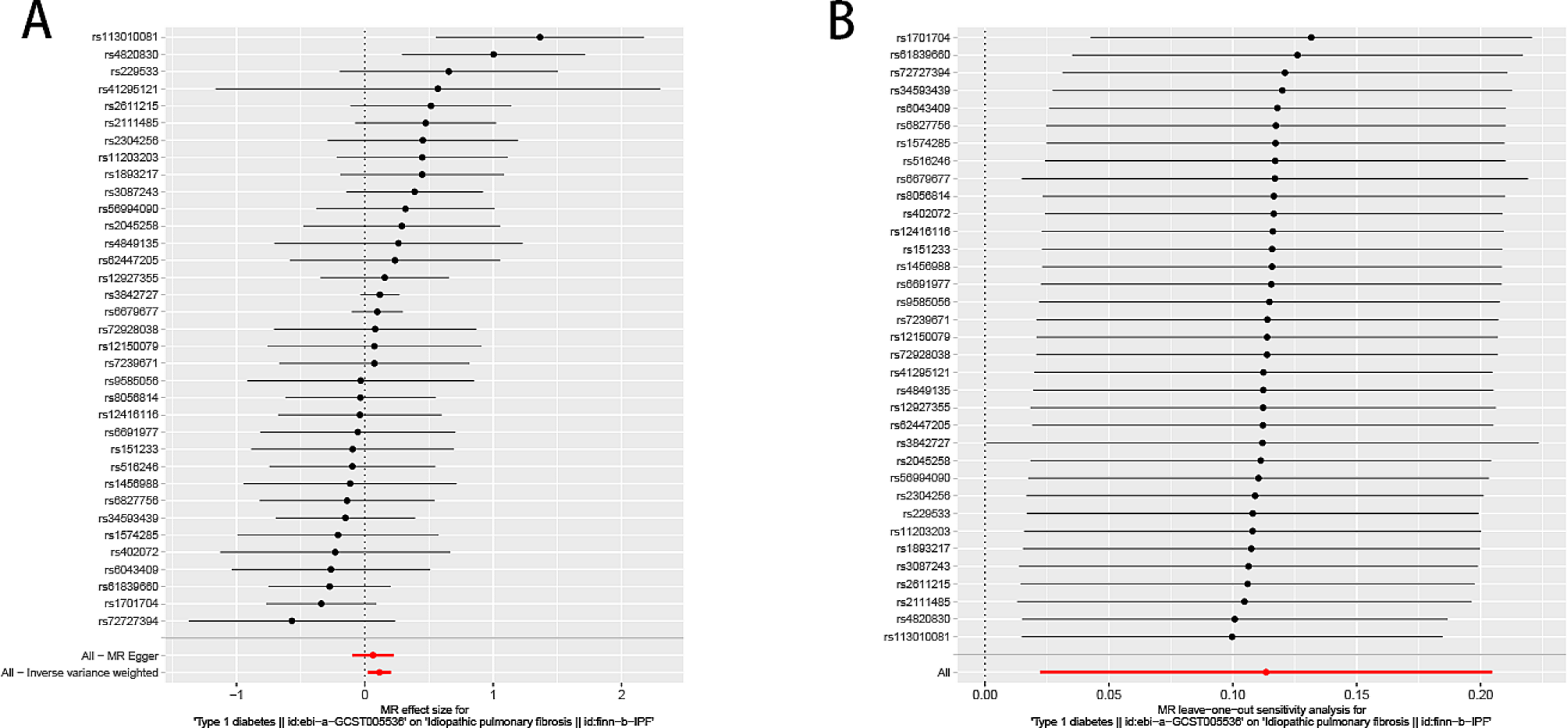
Forest plot of included SNPs in the MR analysis for causal effect of T1D on IPF. **(A)** Meta-analysis of SNPs’ effects on IPF based on IVW method with random effect model. **(B)** Sensitivity analysis by omitting every SNP. T1D, type 1 diabetes; IPF, idiopathic pulmonary fibrosis; IVW, inverse variance weighted

**Fig. 6:**
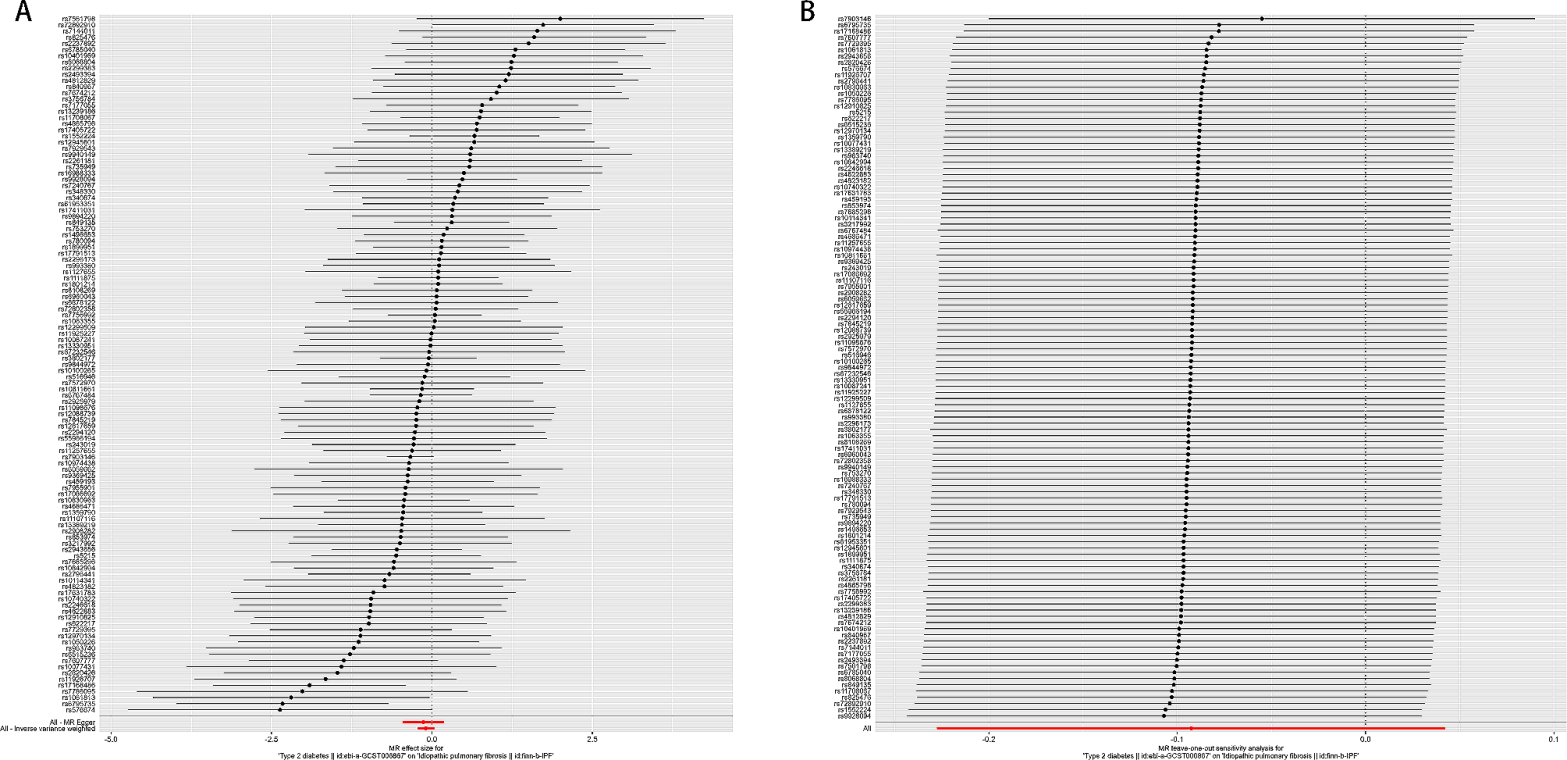
Forest plot of included SNPs in the MR analysis for causal effect of T2D on IPF. **(A)** Meta-analysis of SNPs’ effects on IPF based on IVW method with random effect model. **(B)** Sensitivity analysis by omitting every SNP. T2D, type 2 diabetes; IPF, idiopathic pulmonary fibrosis; IVW, inverse variance weighted

In the sensitivity analysis, the effective IVs used to estimate the effect of T1D on IPF showed no heterogeneity (IVW: I^2^ = 0.14, *P* = 0.232; MR-Egger: I^2^ = 0.15, *P* = 0.217, Table 4). The effective IVs used to estimate the effect of T2D on IPF also showed no heterogeneity (I^2^ = 0, *P* > 0.05, Table 4). The MR-Egger intercepts for the effects of T1D and T2D on IPF were close to 0, with *P*-values of 0.464 and 0.777, respectively, suggesting no substantial horizontal pleiotropic bias (Fig. 4). In addition, the leave-one-out analysis showed that no single SNP in T1D or T2D on IPF affected causal effect estimates (Figs. 5 and 6), whereas effect SNPs in the univariate MR analysis were approximately symmetrical in a funnel plot (Fig. 4). The results of the MVMR heterogeneity test showed no significant heterogeneity and no pleiotropic bias (MR-IVW: *P* = 0.125, MR-Egger: *P* = 0.109; MR-Egger intercept estimate = 0.003, *P* = 0.758). These sensitivity analysis results demonstrated the reliability and statistical power of our univariate and MVMR analyses.

**Table 4:** Sensitivity analyses of MR

Exposure	Heterogeneity test								Pleiotropy test		
	IVW				MR-Egger				MR-Egger intercept		
	Q	Q_df	I^2^	Q_pval	Q	Q_df	I^2^	Q_pval	Intercept	SE	*P*
T1D	39.682	34	0.143	0.232	39.034	33	0.155	0.217	0.0128	0.0173	0.464
T2D	96.322	112	0	0.854	96.242	111	0	0.840	0.003	0.012	0.777

T1D, type 1 diabetes; T2D, type 2 diabetes; IVW, inverse variance weighted; SE, standard error

## Discussion

In this study, we assessed the causal effects of T1D and T2D on IPF using a two-sample univariate MR approach. We identified a causal relationship between T1D and IPF, but no such association was observed between T2D and IPF. Furthermore, the findings of the MVMR analysis were consistent with those of the univariate analysis: namely, that only T1D was associated with an increased risk of IPF. The polygenic genetic predisposition to T2D is certainly greater than that to T1D; therefore, the current study’s finding of an association between T1D and IPF, rather than T2D, looks even more interesting.

Our univariate and multivariate MR analyses indicated that T1D is associated with an average increase in IPF risk of 11.8% and 13.3%, respectively, and genetically-predicted T1D increases the risk of developing IPF. Previous case-control studies have demonstrated a higher prevalence of diabetes among patients with IPF than among healthy individuals or those with other lung diseases [17–21]; however, these studies did not differentiate between specific types of diabetes or only examined patients with T2D. Owing to potential confounding factors in observational studies, recall bias in retrospective cohort studies, and limited sample sizes in some studies, it is not feasible to establish an accurate causal relationship between diabetes and IPF based solely on these types of observational cohort studies.

A large community-based study conducted in the United States demonstrated that patients with T1D exhibit a higher susceptibility to respiratory disease than those with T2D [22], Therefore, we postulated that T1D has a mechanism that impacts the lungs that is unrelated to hyperglycemia. In a UK population-based case-control study including 920 patients with IPF and 3593 controls, significant associations were observed between IPF and diabetes-related exposures, with the strongest association found with insulin use (OR = 2.36, 95% CI = 1.46–3.83, *P* < 0.001), and this association was much stronger than that of IPF and oral hypoglycemic drugs [19].

This study revealed the distinct effects of T1D and T2D on IPF. Patients with T2D do not typically require insulin therapy at disease onset or throughout their lifetime, whereas patients with T1D usually require insulin therapy from the outset because of absolute insulin deficiency [8]. Thus, we deduced that the stronger correlation between IPF and insulin use was attributable to a higher proportion of patients with T1D. However, since the diabetes type was not stratified in the original study and the causality direction remained uncertain, we did not make causal inferences. Future studies should conduct more in-depth analyses of the T1D and T2D subtypes to explore their distinct associations with IPF. Differentiating between the subtypes may provide valuable insights into the specific causal mechanisms underlying the relationship between diabetes and IPF.

Our study revealed no causal relationship between T2D and IPF; however, the results of previous observational studies on the association between these two conditions have been controversial. In a retrospective cohort study using the US Centers for Disease Control and Prevention Multi Cause of Death database, patients with diabetes had a lower overall probability of developing IPF compared to healthy controls (OR = 0.81, 95% CI = 0.79–0.82, *P* < 0.05); moreover, when data were stratified by age, race, and sex, patients with diabetes remained at a reduced risk of developing IPF [23]. This may be attributed to either the occurrence of cardiovascular mortality prior to the development of IPF in patients with diabetes or the prolonged survival of patients with coexisting diabetes and IPF [23]. Another population-based case-control study reported that the incidence of IPF was higher in individuals with T2D compared to non-T2D population (incidence rate ratio = 1.40, 95% CI = 0.76–2.44, *P* = 0.22), although this difference did not reach statistical significance [24]. Notably, the level of glycemic control was not mentioned among participants with T2D in the original study.

Exposure to genetically-determined T2D does not confer an increased risk of IPF; however, the opposite results have been reported in case-control studies, possibly due to the involvement of persistent hyperglycemia in the development of pulmonary fibrosis caused by unstable glycemic control in certain patients with diabetes. Since our study suggests no causal association between T2D and IPF, future research should focus on investigating the characteristics and pathophysiological processes in patients with T2D to elucidate why they may have a relatively lower risk of developing IPF.

IPF is a progressive interstitial lung disease characterized by autoreactive CD4 T cells, and abnormalities in multiple pathways involved in wound healing and inflammation lead to IPF [25, 26]. T1D is an organ-specific autoimmune disease caused by autoimmune-mediated destruction of insulin-producing β-cells in the pancreas [27], and its pathogenesis is intricate, involving a multitude of factors in its development. Characteristics of T1D such as autoimmune processes, autonomic nerve injury, and systemic vascular injury may increase the risk of respiratory disease [22], but the specific mechanisms by which T1D increases the risk of IPF are not clear. A study that included 120 patients with IPF found that the presence of specific anti-modifier protein antibodies (AMPA) in the serum of a substantial proportion of patients with IPF, suggesting that autoimmunity may characterize a subgroup of IPF and potentially exert an influence on disease progression [28]. KAT2B is a transcriptional co-activator that mediates anti-apoptotic effects under metabolic stress conditions and increases cellular resistance to cytotoxic compounds. High KAT2B expression has been identified as a predictive marker for disease progression to transplantation or mortality in patients with IPF, and was observed to be specifically and progressively upregulated in both individuals who progressed to T1D and those who developed islet cell autoantibodies [29]. Elevated KAT2B expression was observed in peripheral blood mononuclear cells (PBMC) of non-obese diabetic (NOD) mice of T1D, preceding the onset of diabetic hyperglycemia [29]. Collectively, this suggests that KAT2B may play a role in the shared pathogenesis of IPF and T1D during disease development.

Citrullination of proteins may contribute to the shared pathogenesis of both T1D and IPF. Citrullination was identified to modify the bioactivity of glucokinase and inhibit glucose-stimulated insulin secretion in the peripheral blood of T1D patients and NOD mice [30]. Autoreactive T cells and autoantibodies targeting citrullinated beta-cell antigens have been detected in the peripheral circulation and immune infiltrates of individuals with T1D, suggesting that citrullination of β-cell antigens may contribute to the pathogenesis of T1D [31–33]. Citrullinated proteins have been detected in bronchoalveolar lavage (BAL) cells from IPF patients, suggesting that the citrullinated pathway is upregulated in IPF [34]. Taken together, post-translational modifications of proteins, particularly citrullination, may contribute to the pathogenesis of IPF and activate immune responses through antibody reactivity [28]. However, limited research has been conducted on the shared pathogenesis of IPF and T1D, and further investigations are required to elucidate their underlying mechanisms.

Hyperglycemia is now known to exert a favorable effect in increasing the risk of IPF, diabetic damage to systemic multi-systems, such as that observed in diabetic nephropathy and diabetic retinopathy, is usually mediated by microvascular injury [35]. The pulmonary interstitium is composed of connective tissue, lymphatic vessels, nerve fibers, and blood vessels, suggesting that diabetes can lead to interstitial lung disease. Both T1D and T2D can affect microvessels through persistent hyperglycemia [35], leading to the development of IPF. However, further research is required to investigate the role of mechanisms other than elevated blood glucose levels in mediating the development of IPF.

In animal models of T1D and T2D, the simulation of clinical hyperglycemia has revealed an increase in DNA double-strand break signals that could not be repaired in a timely manner, resulting in sustained DNA damage [36]. Impaired DNA repair capacity leads to cell senescence, activates the senescence-associated secretory phenotype (including interleukins, inflammatory cytokines, and growth factors), and affects other cell tissues through paracrine signaling, which in turn leads to pulmonary fibrosis [37]. Future research should combine long-term follow-up and clinical case studies of individuals with T1D and IPF to help validate the causal associations and investigate potential biological mechanisms.

Our study presents novel evidence indicating that individuals with a genetic predisposition to T1D were associated with an increased risk of IPF, while no significant association was observed between T2D and IPF. Conflicting results from case-control study results may be due to hyperglycemia resulting from unstable blood glucose control during the development of pulmonary fibrosis. The utilization of diverse hypoglycemic agents could potentially ameliorate pulmonary fibrosis. Recently, several hypoglycemic agents, including metformin, liraglutide, rosiglitazone, and empagliflozin, have been shown to improve pulmonary fibrosis in animal models [38–42].

Currently, apart from metformin, the protective effects of hypoglycemic drugs against pulmonary fibrosis have only been demonstrated in animal and in vitro experiments. Further clinical research is necessary to gain a better understanding of the effects of different types of hypoglycemic drugs on IPF. Additionally, studying the effect of different types of antidiabetic medications on IPF could reveal their potential protective effects and therapeutic strategies.

This study has certain limitations. First, we examined the causal impact of both diabetes types on IPF without exploring the reverse causal effect. When we performed a reverse causality analysis, only six SNPs were screened from the IPF dataset, and these six SNPs did not correspond with the GWAS of T1D and T2D. However, our causal inferences about the two types of diabetes and IPF were reliable; the F-statistics of the IVs we selected were all greater than 10, and the sensitivity analysis did not suggest the existence of significant heterogeneity or pleiotropy. Furthermore, owing to the utilization of GWAS summary-level data instead of individual-level data, we were unable to investigate the impact of variables such as sex, age, and specific types of exposure on outcome or stratify the impact of different glycemic control conditions on IPF. The outcome population in our study consisted of the FinnGen population, although of European origin, represents a distinct and extant group, which may limit the generalization of our conclusions. When analyzing the association between T1D and IPF, researchers should consider exploring factors such as age, sex, specific types of diabetes, diabetes duration, and blood glucose control levels among patients with T1D, and their relationship with IPF development. This would enhance our understanding of the complex relationship between T1D and IPF, and guide clinical management and treatment strategies.

## Conclusion

In summary, our MR study demonstrated that T1D significantly elevates the risk of IPF, whereas no significant association was observed between T2D and IPF. Future studies should consider using larger clinical cohorts or conducting clinical trials to validate our findings to ensure more robust and comprehensive conclusions. Moreover, DM should be considered in future studies on the pathogenesis of IPF, particularly focusing on factors other than hyperglycemia in T1D, and attention should be directed towards exploring the potential antifibrotic effects of hypoglycemic drugs. In clinical practice, patients with T1D should undergo lung imaging for early screening for IPF.

### Electronic supplementary material

Below is the link to the electronic supplementary material.

Supplementary Material 1

## Data Availability

The GWAS summary datasets for T1D (GWAS ID: ebi-a-GCST005536), T2D (GWAS ID: ebi-a-GCST006867), and IPF (GWAS ID: finn-b-IPF) are available through the ieu open gwas project (https://gwas.mrcieu.ac.uk/datasets).

## Abbreviations

IPF: Idiopathic pulmonary fibrosis
T1D: Type 1 diabetes
T2D: Type 2 diabetes
MR: Mendelian randomization
MVMR: Multivariate mendelian randomization
GWAS: Genome-wide association study
SNPs: Single nucleotide polymorphisms
IVW: Inverse variance-weighted
WM: Weighted median
CHF: Congestive heart failure
DM: Diabetes mellitus
COPD: Chronic obstructive pulmonary disease
IVs: Instrumental variables
LASSO: Least absolute shrinkage and selection operator
FVC: Forced vital capacity
