# Supplementary material for: Diabetes mellitus and idiopathic pulmonary fibrosis: a Mendelian randomization study
Source: BMC Pulm Med. 2024 Mar 20;24:142. doi: 10.1186/s12890-024-02961-7 (PMC10953180; doi:10.1186/s12890-024-02961-7)
Supplement: Supplementary file 1 — Supplementary Material 1 [file 12890_2024_2961_MOESM1_ESM.docx]

**SUPPLEMENTAL MATERIAL**

**Diabetes mellitus and idiopathic pulmonary fibrosis: A Mendelian randomization study**

Quou Kang^1, 2^, Jing Ren^1, 2^, Jinpeng Cong^1^, Wencheng Yu^1*^

^1^Department of Pulmonary and Critical Care Medicine, the affiliated hospital of Qingdao University, Qingdao University, Qingdao, China

^2^Medical Department of Qingdao University, Qingdao, China

^*^Correspondence: Wencheng Yu, [ywcheng00@sina.com](mailto:ywcheng00@sina.com)

**Supplementary Materials**

**Supplementary Table 1.** SNPs used as valid instrumental variables for T1D on IPF.

**Supplementary Table 2.** SNPs used as valid instrumental variables for T2D on IPF.

**Supplementary Table 3.** SNPs used as genetic instruments for multivariable Mendelian randomization analysis.

**Supplementary Table 4.** STROBE-MR checklist of recommended items to address in reports of Mendelian randomization studies.

**Supplementary File.** The main code used in the MR analyses.

**Supplementary Table 1.** SNPs used as valid instrumental variables for T1D on IPF.

| **SNP** | **Chr** | **EA** | **OA** | **Position** | **T1D** | | | | | **IPF** | | | **Keep** |
| --- | --- | --- | --- | --- | --- | --- | --- | --- | --- | --- | --- | --- | --- |
|  |  |  |  |  | **Beta** | **SE** | **EAF** | **P** | **F** | **Beta** | **SE** | **P** |  |
| **rs11203203** | **21** | **A** | **G** | **43836186** | **0.149971** | **0.0189963** | **0.197883** | **2.91E-15** | **62.32704479** | **0.0671** | **0.050944669** | **0.1878** | **TRUE** |
| **rs113010081** | **3** | **C** | **T** | **46457412** | **-0.16405** | **0.0300091** | **0.028355** | **4.59E-08** | **29.88453674** | **-0.2239** | **0.067887467** | **0.000973397** | **TRUE** |
| **rs12150079** | **17** | **A** | **G** | **38025417** | **0.114043** | **0.0200075** | **0.186302** | **1.20E-08** | **32.49014245** | **0.0085** | **0.048472852** | **0.8608** | **TRUE** |
| **rs12416116** | **10** | **A** | **C** | **90035654** | **-0.164993** | **0.0209991** | **0.296326** | **3.93E-15** | **61.73474733** | **0.0064** | **0.053568232** | **0.9049** | **TRUE** |
| **rs12927355** | **16** | **T** | **C** | **11194771** | **-0.193949** | **0.0199947** | **0.271565** | **3.02E-22** | **94.09039781** | **-0.03** | **0.049515439** | **0.5446** | **TRUE** |
| **rs1456988** | **14** | **T** | **G** | **98488007** | **-0.111005** | **0.0200008** | **0.542931** | **2.86E-08** | **30.80281079** | **0.0127** | **0.0469784** | **0.786901** | **TRUE** |
| **rs151233** | **16** | **T** | **C** | **28506428** | **0.171008** | **0.0260012** | **0.155751** | **4.80E-11** | **43.25597164** | **-0.0163** | **0.068867465** | **0.8129** | **TRUE** |
| **rs1574285** | **9** | **T** | **G** | **4283137** | **-0.115024** | **0.0190039** | **0.510184** | **1.42E-09** | **36.63460047** | **0.024** | **0.045879741** | **0.6009** | **TRUE** |
| **rs1701704** | **12** | **G** | **T** | **56412487** | **0.222984** | **0.0189986** | **0.201877** | **8.25E-32** | **137.7539958** | **-0.0756** | **0.048834288** | **0.1216** | **TRUE** |
| **rs1893217** | **18** | **G** | **A** | **12809340** | **0.192024** | **0.024003** | **0.119609** | **1.24E-15** | **64** | **0.0858** | **0.062497873** | **0.1698** | **TRUE** |
| **rs2045258** | **6** | **G** | **A** | **126674354** | **0.116983** | **0.0189972** | **0.553115** | **7.37E-10** | **37.91982455** | **0.0172** | **0.04588979** | **0.707801** | **TRUE** |
| **rs2111485** | **2** | **G** | **A** | **163110536** | **0.164993** | **0.0189991** | **0.339257** | **3.81E-18** | **75.41625824** | **0.0782** | **0.046202296** | **0.0905399** | **TRUE** |
| **rs229533** | **22** | **C** | **A** | **37587111** | **0.106969** | **0.0189945** | **0.554113** | **1.79E-08** | **31.71466584** | **0.07** | **0.046424567** | **0.1316** | **TRUE** |
| **rs2304256** | **19** | **A** | **C** | **10475652** | **-0.139032** | **0.0210049** | **0.265775** | **3.61E-11** | **43.81151828** | **-0.0628** | **0.052598796** | **0.2325** | **TRUE** |
| **rs2611215** | **4** | **G** | **A** | **166574267** | **-0.167969** | **0.0249954** | **0.818291** | **1.82E-11** | **45.15835268** | **-0.0866** | **0.053635982** | **0.1064** | **TRUE** |
| **rs3087243** | **2** | **A** | **G** | **204738919** | **-0.178051** | **0.0190054** | **0.36901** | **7.36E-21** | **87.76771775** | **-0.0691** | **0.048378541** | **0.1532** | **TRUE** |
| **rs3184504** | **12** | **C** | **T** | **111884608** | **-0.266051** | **0.0190036** | **0.852636** | **1.56E-44** | **196.000884** | **-0.0885** | **0.046388986** | **0.0564196** | **FALSE** |
| **rs34593439** | **15** | **A** | **G** | **79234957** | **-0.246028** | **0.0330038** | **0.096446** | **9.02E-14** | **55.57010009** | **0.0372** | **0.068210742** | **0.5855** | **TRUE** |
| **rs3842727** | **11** | **T** | **G** | **2184848** | **0.686966** | **0.0229989** | **0.642372** | **4.89E-196** | **892.1879561** | **0.0802** | **0.05318929** | **0.1316** | **TRUE** |
| **rs402072** | **19** | **C** | **T** | **47219122** | **-0.142025** | **0.0260045** | **0.098043** | **4.72E-08** | **29.82858008** | **0.0327** | **0.064943221** | **0.614601** | **TRUE** |
| **rs41295121** | **10** | **T** | **C** | **6129643** | **-0.652965** | **0.111994** | **0.025759** | **5.53E-09** | **33.99306255** | **-0.3716** | **0.577052579** | **0.5196** | **TRUE** |
| **rs4820830** | **22** | **T** | **C** | **30531091** | **-0.134968** | **0.0189955** | **0.722843** | **1.20E-12** | **50.48474486** | **-0.1354** | **0.049108881** | **0.00583096** | **TRUE** |
| **rs4849135** | **2** | **G** | **T** | **111615079** | **0.114962** | **0.0209931** | **0.692891** | **4.35E-08** | **29.98855034** | **0.0301** | **0.056713428** | **0.5956** | **TRUE** |
| **rs516246** | **19** | **T** | **C** | **49206172** | **0.142947** | **0.018993** | **0.320687** | **5.22E-14** | **56.64517909** | **-0.0139** | **0.047119924** | **0.768** | **TRUE** |
| **rs56994090** | **14** | **C** | **T** | **101306447** | **-0.12897** | **0.0189956** | **0.369209** | **1.13E-11** | **46.09686261** | **-0.0408** | **0.045750155** | **0.3725** | **TRUE** |
| **rs6043409** | **20** | **G** | **A** | **1616206** | **0.126017** | **0.0200027** | **0.797125** | **2.98E-10** | **39.6899937** | **-0.0333** | **0.04972969** | **0.5031** | **TRUE** |
| **rs61839660** | **10** | **T** | **C** | **6094697** | **-0.471925** | **0.0359943** | **0.028155** | **2.84E-39** | **171.9010403** | **0.1293** | **0.114430492** | **0.2585** | **TRUE** |
| **rs62447205** | **7** | **G** | **A** | **50465830** | **-0.116983** | **0.020997** | **0.226238** | **2.53E-08** | **31.0406647** | **-0.0275** | **0.048904792** | **0.5739** | **TRUE** |
| **rs6679677** | **1** | **A** | **C** | **114303808** | **0.635995** | **0.0269998** | **0.025759** | **1.10E-122** | **554.8636935** | **0.0618** | **0.064782069** | **0.3401** | **TRUE** |
| **rs6691977** | **1** | **C** | **T** | **200814959** | **0.126016** | **0.0230029** | **0.424521** | **4.30E-08** | **30.011396** | **-0.0069** | **0.048861623** | **0.8877** | **TRUE** |
| **rs6827756** | **4** | **C** | **T** | **123184411** | **-0.131028** | **0.0190041** | **0.591653** | **5.40E-12** | **47.53720135** | **0.0181** | **0.045565874** | **0.6912** | **TRUE** |
| **rs7239671** | **18** | **G** | **A** | **67523260** | **0.120978** | **0.0179967** | **0.464058** | **1.79E-11** | **45.18840856** | **0.009** | **0.045766162** | **0.8441** | **TRUE** |
| **rs72727394** | **15** | **T** | **C** | **38847022** | **0.138021** | **0.0220034** | **0.13778** | **3.55E-10** | **39.34692084** | **-0.0784** | **0.056572753** | **0.1658** | **TRUE** |
| **rs72928038** | **6** | **A** | **G** | **90976768** | **0.179985** | **0.0239981** | **0.071486** | **6.38E-14** | **56.24953121** | **0.0145** | **0.072370178** | **0.8412** | **TRUE** |
| **rs8056814** | **16** | **A** | **G** | **75252327** | **0.27801** | **0.0310012** | **0.180911** | **3.03E-19** | **80.41995498** | **-0.0095** | **0.082931561** | **0.9088** | **TRUE** |
| **rs9585056** | **13** | **T** | **C** | **100081766** | **-0.116004** | **0.0210007** | **0.717652** | **3.32E-08** | **30.5125418** | **0.0037** | **0.05229986** | **0.9436** | **TRUE** |

rs3184504 were removed for its correlation with smoking.

SNPs, single-nucleotide polymorphisms. T1D, type 1 diabetes. IPF, idiopathic pulmonary fibrosis. Chr, chromosome. EA, effect allele. OA, other allele. SE, standard error. EAF, effect allele frequency.

**Supplementary Table 2.** SNPs used as valid instrumental variables for T2D on IPF.

| **SNP** | **Chr** | **EA** | **OA** | **Position** | **T2D** | | | | | **IPF** | | | **Keep** |
| --- | --- | --- | --- | --- | --- | --- | --- | --- | --- | --- | --- | --- | --- |
|  |  |  |  |  | **Beta** | **SE** | **EAF** | **P** | **F** | **Beta** | **SE** | **P** |  |
| **rs10077431** | **5** | **A** | **C** | **112927686** | **-0.0487** | **0.0089** | **0.214668** | **4.75E-08** | **29.94180028** | **0.0688** | **0.059934289** | **0.251** | **TRUE** |
| **rs10087241** | **8** | **A** | **G** | **30863722** | **-0.0475** | **0.008** | **0.594893** | **2.80E-09** | **35.25390625** | **0.001** | **0.045589748** | **0.9825** | **TRUE** |
| **rs10100265** | **8** | **C** | **A** | **10633159** | **-0.0491** | **0.0079** | **0.61049** | **6.29E-10** | **38.62858516** | **0.0042** | **0.061895476** | **0.9459** | **TRUE** |
| **rs10114341** | **9** | **C** | **T** | **96919182** | **-0.0409** | **0.0072** | **0.440754** | **1.15E-08** | **32.26871142** | **0.0302** | **0.045925096** | **0.5108** | **TRUE** |
| **rs10401969** | **19** | **C** | **T** | **19407718** | **0.0921** | **0.0133** | **0.0765858** | **4.13E-12** | **47.95302165** | **0.1178** | **0.094302018** | **0.2116** | **TRUE** |
| **rs1050226** | **6** | **G** | **A** | **7281654** | **-0.0491** | **0.0074** | **0.406792** | **3.34E-11** | **44.02501826** | **0.0561** | **0.047057592** | **0.2332** | **TRUE** |
| **rs1061813** | **5** | **A** | **G** | **14847331** | **-0.0429** | **0.0073** | **0.537119** | **3.37E-09** | **34.5357478** | **0.094** | **0.047175461** | **0.0463095** | **TRUE** |
| **rs1063355** | **6** | **G** | **T** | **32627714** | **0.0709** | **0.0079** | **0.602436** | **3.72E-19** | **80.54494472** | **0.0032** | **0.048505318** | **0.9474** | **TRUE** |
| **rs10740322** | **10** | **A** | **G** | **71485458** | **0.0477** | **0.0085** | **0.687104** | **2.11E-08** | **31.49190311** | **-0.0453** | **0.052047024** | **0.3841** | **TRUE** |
| **rs10811661** | **9** | **C** | **T** | **22134094** | **-0.1569** | **0.0098** | **0.173605** | **4.13E-58** | **256.3266347** | **0.0245** | **0.065039523** | **0.706401** | **TRUE** |
| **rs10830963** | **11** | **G** | **C** | **92708710** | **0.0909** | **0.008** | **0.275768** | **5.85E-30** | **129.1064063** | **-0.0394** | **0.047638313** | **0.4082** | **TRUE** |
| **rs10842994** | **12** | **T** | **C** | **27965150** | **-0.0755** | **0.0091** | **0.197025** | **1.02E-16** | **68.83528559** | **0.045** | **0.059926516** | **0.452701** | **TRUE** |
| **rs10974438** | **9** | **C** | **A** | **4291928** | **0.0591** | **0.0075** | **0.351215** | **3.01E-15** | **62.0944** | **-0.0211** | **0.046974369** | **0.653301** | **TRUE** |
| **rs11098676** | **4** | **C** | **T** | **123833154** | **0.054** | **0.0096** | **0.787639** | **2.03E-08** | **31.640625** | **-0.0122** | **0.059226646** | **0.8368** | **TRUE** |
| **rs11107116** | **12** | **T** | **G** | **93978504** | **0.0467** | **0.0085** | **0.219714** | **3.75E-08** | **30.18532872** | **-0.0216** | **0.052822355** | **0.6826** | **TRUE** |
| **rs1111875** | **10** | **T** | **C** | **94462882** | **-0.0948** | **0.0072** | **0.408262** | **3.61E-39** | **173.3611111** | **-0.0092** | **0.045626397** | **0.8402** | **TRUE** |
| **rs11257655** | **10** | **T** | **C** | **12307894** | **0.0737** | **0.0087** | **0.206773** | **1.97E-17** | **71.76231999** | **-0.0227** | **0.05202512** | **0.662599** | **TRUE** |
| **rs1127655** | **1** | **T** | **C** | **117530507** | **-0.0438** | **0.0079** | **0.529064** | **2.47E-08** | **30.7393046** | **-0.0043** | **0.046234331** | **0.9259** | **TRUE** |
| **rs11708067** | **3** | **G** | **A** | **123065778** | **-0.0965** | **0.0086** | **0.23899** | **5.93E-29** | **125.9092753** | **-0.0719** | **0.060932175** | **0.238** | **TRUE** |
| **rs11925227** | **3** | **A** | **G** | **170766618** | **-0.0534** | **0.0095** | **0.183439** | **2.25E-08** | **31.59623269** | **0.0004** | **0.054093376** | **0.9941** | **TRUE** |
| **rs11926707** | **3** | **C** | **T** | **46925539** | **0.0463** | **0.0082** | **0.625556** | **1.69E-08** | **31.88117192** | **-0.0767** | **0.048288134** | **0.1122** | **TRUE** |
| **rs12088739** | **1** | **G** | **A** | **51506886** | **-0.0884** | **0.013** | **0.0898481** | **9.79E-12** | **46.24** | **0.0216** | **0.096618529** | **0.8231** | **TRUE** |
| **rs12299509** | **12** | **G** | **A** | **4406281** | **0.0467** | **0.0073** | **0.478622** | **2.09E-10** | **40.92493901** | **0.0013** | **0.047793642** | **0.9783** | **TRUE** |
| **rs12617659** | **2** | **T** | **C** | **121309759** | **-0.0685** | **0.0103** | **0.147238** | **2.83E-11** | **44.22895655** | **0.017** | **0.064210558** | **0.791199** | **TRUE** |
| **rs12910825** | **15** | **G** | **A** | **91511260** | **0.0517** | **0.0074** | **0.360391** | **2.16E-12** | **48.81099343** | **-0.0506** | **0.047140743** | **0.2831** | **TRUE** |
| **rs12945601** | **17** | **C** | **T** | **17653411** | **-0.048** | **0.008** | **0.613603** | **1.72E-09** | **36** | **-0.0316** | **0.045871688** | **0.4909** | **TRUE** |
| **rs12970134** | **18** | **A** | **G** | **57884750** | **0.0555** | **0.008** | **0.26512** | **5.31E-12** | **48.12890625** | **-0.0619** | **0.057752128** | **0.2838** | **TRUE** |
| **rs13234269** | **7** | **A** | **T** | **130429186** | **-0.0583** | **0.0078** | **0.492515** | **6.98E-14** | **55.86604208** | **0.0045** | **0.045317175** | **0.9209** | **FALSE** |
| **rs13239186** | **7** | **T** | **C** | **117510621** | **0.0539** | **0.0085** | **0.302029** | **2.70E-10** | **40.21051903** | **0.0411** | **0.047530588** | **0.3872** | **TRUE** |
| **rs13330951** | **16** | **G** | **A** | **69563842** | **-0.0456** | **0.0081** | **0.488314** | **1.54E-08** | **31.69272977** | **0.001** | **0.04777403** | **0.9833** | **TRUE** |
| **rs13389219** | **2** | **T** | **C** | **165528876** | **-0.0722** | **0.0074** | **0.394368** | **2.11E-22** | **95.19430241** | **0.0338** | **0.048084515** | **0.4821** | **TRUE** |
| **rs1359790** | **13** | **A** | **G** | **80717156** | **-0.0796** | **0.008** | **0.2867** | **2.80E-23** | **99.0025** | **0.0357** | **0.050031024** | **0.475501** | **TRUE** |
| **rs1496653** | **3** | **G** | **A** | **23454790** | **-0.0769** | **0.0088** | **0.204782** | **2.57E-18** | **76.3637655** | **-0.0143** | **0.04919564** | **0.771299** | **TRUE** |
| **rs1552224** | **11** | **C** | **A** | **72433098** | **-0.1034** | **0.0101** | **0.15421** | **8.64E-25** | **104.8089403** | **-0.0685** | **0.053379555** | **0.1994** | **TRUE** |
| **rs16988333** | **22** | **G** | **A** | **30552813** | **-0.0745** | **0.013** | **0.0904035** | **9.17E-09** | **32.84171598** | **-0.0369** | **0.082225362** | **0.6536** | **TRUE** |
| **rs17086692** | **4** | **T** | **G** | **53134293** | **-0.0467** | **0.0084** | **0.313426** | **2.48E-08** | **30.90830499** | **0.0194** | **0.049072866** | **0.692599** | **TRUE** |
| **rs17168486** | **7** | **T** | **C** | **14898282** | **0.0742** | **0.0094** | **0.173603** | **2.18E-15** | **62.30918968** | **-0.1415** | **0.056913259** | **0.0129101** | **TRUE** |
| **rs17405722** | **17** | **A** | **G** | **40542501** | **0.087** | **0.0146** | **0.0741526** | **2.28E-09** | **35.50853819** | **0.0605** | **0.075281668** | **0.4216** | **TRUE** |
| **rs17411031** | **8** | **G** | **C** | **19852310** | **-0.045** | **0.0081** | **0.261729** | **3.04E-08** | **30.86419753** | **-0.0143** | **0.052795128** | **0.7865** | **TRUE** |
| **rs1758632** | **9** | **G** | **C** | **34025640** | **0.0491** | **0.0081** | **0.623407** | **1.36E-09** | **36.74455114** | **-0.0537** | **0.045731813** | **0.2403** | **FALSE** |
| **rs17631783** | **17** | **T** | **C** | **61687600** | **-0.0487** | **0.0089** | **0.26346** | **3.95E-08** | **29.94180028** | **0.0444** | **0.055058073** | **0.42** | **TRUE** |
| **rs17791513** | **9** | **G** | **A** | **81905590** | **-0.1027** | **0.0148** | **0.0605985** | **4.61E-12** | **48.1523466** | **-0.0145** | **0.069699107** | **0.8352** | **TRUE** |
| **rs1801214** | **4** | **T** | **C** | **6303022** | **0.0903** | **0.0074** | **0.599585** | **5.52E-34** | **148.9059533** | **0.0087** | **0.046065077** | **0.8502** | **TRUE** |
| **rs1899951** | **3** | **T** | **C** | **12394840** | **-0.1118** | **0.0109** | **0.123288** | **1.64E-24** | **105.2036024** | **-0.0166** | **0.060617156** | **0.7842** | **TRUE** |
| **rs2058913** | **15** | **T** | **A** | **63831869** | **-0.0491** | **0.0078** | **0.565008** | **3.26E-10** | **39.62541091** | **0.0385** | **0.046057048** | **0.4032** | **FALSE** |
| **rs2237892** | **11** | **T** | **C** | **2839751** | **-0.096** | **0.0157** | **0.0624896** | **8.75E-10** | **37.38894073** | **-0.145** | **0.104531854** | **0.1654** | **TRUE** |
| **rs2246618** | **6** | **T** | **C** | **31478986** | **0.0513** | **0.0084** | **0.307253** | **1.20E-09** | **37.29719388** | **-0.049** | **0.053286051** | **0.3578** | **TRUE** |
| **rs2261181** | **12** | **T** | **C** | **66212318** | **0.0985** | **0.0118** | **0.09647** | **9.18E-17** | **69.68004884** | **0.0587** | **0.08747717** | **0.5022** | **TRUE** |
| **rs2294120** | **8** | **G** | **A** | **146003567** | **-0.0443** | **0.0079** | **0.455879** | **1.62E-08** | **31.44512097** | **0.0117** | **0.045970319** | **0.7991** | **TRUE** |
| **rs2296173** | **1** | **G** | **A** | **39913351** | **0.065** | **0.0087** | **0.212011** | **7.66E-14** | **55.81979125** | **0.0072** | **0.057586296** | **0.9005** | **TRUE** |
| **rs2299383** | **7** | **T** | **C** | **103418846** | **0.0412** | **0.0073** | **0.423455** | **1.49E-08** | **31.85288047** | **0.0508** | **0.045679808** | **0.2661** | **TRUE** |
| **rs243019** | **2** | **C** | **T** | **60585806** | **0.0566** | **0.0071** | **0.455831** | **2.29E-15** | **63.55008927** | **-0.0161** | **0.045818944** | **0.725301** | **TRUE** |
| **rs2493394** | **1** | **G** | **A** | **120471224** | **0.073** | **0.0113** | **0.107338** | **1.15E-10** | **41.73388676** | **0.0874** | **0.066101717** | **0.1861** | **TRUE** |
| **rs2796441** | **9** | **A** | **G** | **84308948** | **-0.0715** | **0.0073** | **0.416458** | **1.96E-22** | **95.93263276** | **0.0475** | **0.046106014** | **0.3029** | **TRUE** |
| **rs2820426** | **1** | **G** | **A** | **219660535** | **0.0521** | **0.0073** | **0.610058** | **1.30E-12** | **50.93657347** | **-0.0768** | **0.046965755** | **0.102** | **TRUE** |
| **rs2867125** | **2** | **C** | **T** | **622827** | **0.0601** | **0.0096** | **0.827825** | **4.33E-10** | **39.19281684** | **0.0278** | **0.061947562** | **0.6536** | **FALSE** |
| **rs2908282** | **7** | **A** | **G** | **44248828** | **0.0552** | **0.0094** | **0.177395** | **4.25E-09** | **34.48438207** | **-0.0264** | **0.074341153** | **0.7225** | **TRUE** |
| **rs2925979** | **16** | **C** | **T** | **81534790** | **-0.0534** | **0.0078** | **0.70085** | **9.06E-12** | **46.86982249** | **0.0105** | **0.048643541** | **0.8291** | **TRUE** |
| **rs2943656** | **2** | **G** | **A** | **227121918** | **0.0902** | **0.0074** | **0.635133** | **6.70E-34** | **148.5763331** | **-0.0495** | **0.046626057** | **0.2884** | **TRUE** |
| **rs3217992** | **9** | **T** | **C** | **22003223** | **0.0527** | **0.0073** | **0.369901** | **7.23E-13** | **52.11653218** | **-0.0261** | **0.046793303** | **0.577** | **TRUE** |
| **rs340874** | **1** | **C** | **T** | **214159256** | **0.0626** | **0.0073** | **0.563904** | **8.41E-18** | **73.5364984** | **0.0227** | **0.04630878** | **0.624001** | **TRUE** |
| **rs348330** | **1** | **A** | **G** | **229672955** | **-0.0487** | **0.0081** | **0.633451** | **1.86E-09** | **36.14830056** | **-0.0196** | **0.048107839** | **0.683701** | **TRUE** |
| **rs3756784** | **6** | **G** | **T** | **131950233** | **0.0505** | **0.0091** | **0.185838** | **2.59E-08** | **30.7964014** | **0.0464** | **0.055436957** | **0.4026** | **TRUE** |
| **rs3802177** | **8** | **A** | **G** | **118185025** | **-0.1217** | **0.008** | **0.311281** | **2.32E-52** | **231.4201563** | **0.0061** | **0.04680184** | **0.8963** | **TRUE** |
| **rs459193** | **5** | **G** | **A** | **55806751** | **0.0711** | **0.0083** | **0.745338** | **8.81E-18** | **73.38089708** | **-0.0268** | **0.04862163** | **0.5815** | **TRUE** |
| **rs4622883** | **3** | **G** | **A** | **152188290** | **-0.0435** | **0.0078** | **0.508544** | **3.02E-08** | **31.10207101** | **0.0418** | **0.047146965** | **0.3753** | **TRUE** |
| **rs4686471** | **3** | **C** | **T** | **187740899** | **0.0534** | **0.0081** | **0.609775** | **4.28E-11** | **43.46227709** | **-0.0236** | **0.046883451** | **0.6147** | **TRUE** |
| **rs4812829** | **20** | **A** | **G** | **42989267** | **0.0532** | **0.0095** | **0.160871** | **2.44E-08** | **31.36** | **0.0611** | **0.056076864** | **0.2759** | **TRUE** |
| **rs4823182** | **22** | **G** | **A** | **44377442** | **0.0482** | **0.0077** | **0.335748** | **3.36E-10** | **39.18434812** | **-0.0356** | **0.045592264** | **0.4349** | **TRUE** |
| **rs4865796** | **5** | **A** | **G** | **53272664** | **0.053** | **0.0078** | **0.69309** | **1.33E-11** | **46.17028271** | **0.0372** | **0.048407026** | **0.4422** | **TRUE** |
| **rs516946** | **8** | **C** | **T** | **41519248** | **0.0824** | **0.0085** | **0.760633** | **3.16E-22** | **93.97591696** | **-0.0093** | **0.056257945** | **0.8687** | **TRUE** |
| **rs5215** | **11** | **T** | **C** | **17408630** | **-0.0678** | **0.0073** | **0.639941** | **2.09E-20** | **86.26083693** | **0.0377** | **0.0457095** | **0.4095** | **TRUE** |
| **rs55966194** | **20** | **G** | **C** | **45599090** | **-0.0526** | **0.0088** | **0.281312** | **2.25E-09** | **35.72778926** | **0.0147** | **0.055523247** | **0.791199** | **TRUE** |
| **rs576674** | **13** | **A** | **G** | **33554302** | **-0.0654** | **0.0097** | **0.832515** | **1.79E-11** | **45.45817834** | **0.1548** | **0.078894632** | **0.0497496** | **TRUE** |
| **rs6059662** | **20** | **G** | **A** | **32675727** | **0.0446** | **0.0079** | **0.663176** | **1.51E-08** | **31.87245634** | **-0.0161** | **0.054674552** | **0.768399** | **TRUE** |
| **rs61953351** | **12** | **T** | **G** | **121456616** | **-0.07** | **0.0091** | **0.249902** | **1.98E-14** | **59.17159763** | **-0.0232** | **0.050417036** | **0.6454** | **TRUE** |
| **rs622217** | **6** | **C** | **T** | **160766770** | **-0.0485** | **0.0077** | **0.483932** | **3.13E-10** | **39.67363805** | **0.0477** | **0.045521055** | **0.2947** | **TRUE** |
| **rs6494307** | **15** | **G** | **C** | **62394690** | **-0.0443** | **0.0078** | **0.426225** | **1.67E-08** | **32.25657462** | **-0.0132** | **0.045719934** | **0.7728** | **FALSE** |
| **rs6515236** | **20** | **C** | **A** | **22435749** | **-0.0504** | **0.0091** | **0.24933** | **3.34E-08** | **30.67455621** | **0.0644** | **0.056290902** | **0.2526** | **TRUE** |
| **rs67232546** | **11** | **T** | **C** | **128398938** | **0.0596** | **0.0096** | **0.209222** | **4.66E-10** | **38.54340278** | **-0.0026** | **0.064407968** | **0.9678** | **TRUE** |
| **rs6767484** | **3** | **G** | **A** | **185520578** | **0.1209** | **0.0076** | **0.312261** | **2.70E-56** | **253.061115** | **-0.021** | **0.049167787** | **0.6693** | **TRUE** |
| **rs6785040** | **3** | **C** | **T** | **63897915** | **-0.0633** | **0.0111** | **0.15066** | **1.26E-08** | **32.52081812** | **-0.0826** | **0.055050453** | **0.1335** | **TRUE** |
| **rs6795735** | **3** | **T** | **C** | **64705365** | **-0.0558** | **0.0073** | **0.410912** | **1.63E-14** | **58.42822293** | **0.1298** | **0.047096956** | **0.005851** | **TRUE** |
| **rs6878122** | **5** | **A** | **G** | **76427311** | **-0.0564** | **0.0079** | **0.681791** | **1.19E-12** | **50.96875501** | **-0.0042** | **0.054347118** | **0.9384** | **TRUE** |
| **rs6960043** | **7** | **C** | **T** | **15052860** | **0.064** | **0.0071** | **0.521837** | **3.61E-19** | **81.2537195** | **0.0048** | **0.046623061** | **0.918** | **TRUE** |
| **rs7144011** | **14** | **T** | **G** | **79940383** | **0.0482** | **0.0085** | **0.221063** | **1.64E-08** | **32.15557093** | **0.0791** | **0.053124613** | **0.1365** | **TRUE** |
| **rs7177055** | **15** | **A** | **G** | **77832762** | **0.0647** | **0.0079** | **0.718289** | **2.75E-16** | **67.0740266** | **0.0507** | **0.049334385** | **0.3041** | **TRUE** |
| **rs7240767** | **18** | **C** | **T** | **7070642** | **0.0451** | **0.0081** | **0.383677** | **2.16E-08** | **31.00152416** | **0.0193** | **0.046668669** | **0.679201** | **TRUE** |
| **rs72802358** | **16** | **C** | **G** | **75243657** | **-0.1168** | **0.0133** | **0.101594** | **1.97E-18** | **77.12273164** | **-0.0068** | **0.076858795** | **0.9295** | **TRUE** |
| **rs72892910** | **6** | **T** | **G** | **50816887** | **0.0648** | **0.0099** | **0.172394** | **6.43E-11** | **42.84297521** | **0.1123** | **0.056919471** | **0.0484998** | **TRUE** |
| **rs735949** | **4** | **C** | **T** | **185716232** | **-0.0711** | **0.0106** | **0.14115** | **1.95E-11** | **44.99118904** | **-0.0414** | **0.075388735** | **0.5829** | **TRUE** |
| **rs753270** | **10** | **C** | **T** | **80964975** | **0.0528** | **0.0079** | **0.583535** | **2.70E-11** | **44.66976446** | **0.0125** | **0.046215915** | **0.786799** | **TRUE** |
| **rs7561798** | **2** | **G** | **A** | **228973660** | **0.04** | **0.0072** | **0.482171** | **2.79E-08** | **30.86419753** | **0.08** | **0.045685768** | **0.07993** | **TRUE** |
| **rs7572970** | **2** | **G** | **A** | **161136656** | **0.059** | **0.0087** | **0.722047** | **1.39E-11** | **45.99022328** | **-0.0087** | **0.056722516** | **0.8781** | **TRUE** |
| **rs7607777** | **2** | **T** | **G** | **43629931** | **-0.137** | **0.0125** | **0.105906** | **9.40E-28** | **120.1216** | **0.1884** | **0.102834389** | **0.0669407** | **TRUE** |
| **rs7674212** | **4** | **T** | **G** | **103988899** | **-0.0465** | **0.0075** | **0.408864** | **6.18E-10** | **38.44** | **-0.047** | **0.046180607** | **0.3088** | **TRUE** |
| **rs7685296** | **4** | **T** | **C** | **153254121** | **-0.0511** | **0.0081** | **0.279365** | **2.32E-10** | **39.79896357** | **0.0303** | **0.049898933** | **0.5437** | **TRUE** |
| **rs7729395** | **5** | **T** | **C** | **102100576** | **0.1373** | **0.016** | **0.0509409** | **1.10E-17** | **73.63785156** | **-0.1522** | **0.09947273** | **0.126** | **TRUE** |
| **rs7756992** | **6** | **G** | **A** | **20679709** | **0.1297** | **0.0078** | **0.266896** | **6.00E-62** | **276.4972058** | **0.0061** | **0.048300382** | **0.8995** | **TRUE** |
| **rs7786095** | **7** | **G** | **A** | **156983847** | **-0.0743** | **0.0129** | **0.10386** | **9.64E-09** | **33.174028** | **0.15** | **0.097724061** | **0.1248** | **TRUE** |
| **rs780094** | **2** | **C** | **T** | **27741237** | **0.0692** | **0.0074** | **0.612845** | **5.16E-21** | **87.4477721** | **0.0106** | **0.047743912** | **0.8243** | **TRUE** |
| **rs7845219** | **8** | **C** | **T** | **95937502** | **-0.0422** | **0.0072** | **0.492786** | **4.54E-09** | **34.35262346** | **0.0104** | **0.045371787** | **0.8187** | **TRUE** |
| **rs7903146** | **10** | **T** | **C** | **114758349** | **0.3059** | **0.0077** | **0.291585** | **1.00E-200** | **1578.256198** | **-0.1026** | **0.057451088** | **0.0741208** | **TRUE** |
| **rs7929543** | **11** | **C** | **A** | **49351026** | **0.0828** | **0.0138** | **0.0831599** | **2.20E-09** | **36** | **0.0507** | **0.091064631** | **0.5777** | **TRUE** |
| **rs7955901** | **12** | **T** | **C** | **71433293** | **-0.0444** | **0.0072** | **0.556668** | **7.16E-10** | **38.02777778** | **0.0182** | **0.047483001** | **0.701501** | **TRUE** |
| **rs8068804** | **17** | **A** | **G** | **3985864** | **0.0587** | **0.0078** | **0.325097** | **4.41E-14** | **56.63527285** | **0.0727** | **0.049783343** | **0.1442** | **TRUE** |
| **rs8108269** | **19** | **G** | **T** | **46158513** | **0.0644** | **0.0079** | **0.281015** | **3.11E-16** | **66.45345297** | **0.005** | **0.048804596** | **0.9184** | **TRUE** |
| **rs825476** | **12** | **T** | **C** | **124568456** | **0.0524** | **0.0073** | **0.580549** | **6.80E-13** | **51.52486395** | **0.0835** | **0.046473593** | **0.0723802** | **TRUE** |
| **rs840967** | **2** | **A** | **C** | **65701757** | **-0.0497** | **0.008** | **0.605922** | **5.44E-10** | **38.59515625** | **-0.0522** | **0.045790794** | **0.2543** | **TRUE** |
| **rs849135** | **7** | **A** | **G** | **28196413** | **-0.0999** | **0.0072** | **0.499052** | **1.04E-43** | **192.515625** | **-0.0309** | **0.04601616** | **0.5019** | **TRUE** |
| **rs853974** | **6** | **C** | **T** | **127068983** | **-0.0601** | **0.0088** | **0.737586** | **7.86E-12** | **46.64269112** | **0.0292** | **0.051231026** | **0.5687** | **TRUE** |
| **rs9369425** | **6** | **A** | **G** | **43810974** | **-0.0546** | **0.0085** | **0.708185** | **1.13E-10** | **41.2617301** | **0.0205** | **0.049196572** | **0.676901** | **TRUE** |
| **rs963740** | **13** | **T** | **A** | **51096095** | **-0.0479** | **0.0086** | **0.294299** | **2.23E-08** | **31.02230936** | **0.0583** | **0.056192434** | **0.2995** | **TRUE** |
| **rs9844972** | **3** | **C** | **G** | **150097635** | **0.0956** | **0.0148** | **0.0697229** | **1.03E-10** | **41.72461651** | **-0.0057** | **0.100120846** | **0.9546** | **TRUE** |
| **rs9894220** | **17** | **G** | **A** | **46989154** | **-0.0585** | **0.0079** | **0.43374** | **1.52E-13** | **54.83496235** | **-0.0182** | **0.046531659** | **0.6957** | **TRUE** |
| **rs9928094** | **16** | **G** | **A** | **53799905** | **0.1045** | **0.0072** | **0.42602** | **3.59E-47** | **210.6529707** | **0.0495** | **0.045905602** | **0.2809** | **TRUE** |
| **rs993380** | **4** | **G** | **A** | **83584496** | **-0.0507** | **0.0081** | **0.66555** | **4.59E-10** | **39.17832647** | **-0.0056** | **0.046724102** | **0.9046** | **TRUE** |
| **rs9940149** | **16** | **A** | **G** | **300641** | **-0.058** | **0.0095** | **0.178556** | **9.29E-10** | **37.27423823** | **-0.0346** | **0.074603132** | **0.6428** | **TRUE** |

rs13234269, rs1758632, rs2058913 and rs6494307 were removed for being palindromic with intermediate allele frequencies. rs3184504 were removed for its correlation with smoking.

SNPs, single-nucleotide polymorphisms. T2D, type 2 diabetes. IPF, idiopathic pulmonary fibrosis. Chr, chromosome. EA, effect allele. OA, other allele. SE, standard error. EAF, effect allele frequency.

**Supplementary Table 3.** SNPs used as genetic instruments for multivariable Mendelian randomization analysis.

| **SNP** | **Type 1 diabetes** | | | **Type 2 diabetes** | | | **Idiopathic pulmonary fibrosis** | | |
| --- | --- | --- | --- | --- | --- | --- | --- | --- | --- |
|  | **Beta** | **SE** | **P** | **Beta** | **SE** | **P** | **Beta** | **SE** | **P** |
| **rs10100265** | **0.039989** | **0.0189948** | **0.0352687** | **-0.0491** | **0.0079** | **6.29E-10** | **0.0042** | **0.0621** | **0.9459** |
| **rs10401969** | **0.061001** | **0.0340006** | **0.0727947** | **0.0921** | **0.0133** | **4.13E-12** | **0.1178** | **0.0943** | **0.2116** |
| **rs10811661** | **0.0289761** | **0.0239802** | **0.226919** | **-0.1569** | **0.0098** | **4.13E-58** | **0.0245** | **0.0651** | **0.706401** |
| **rs10974438** | **0.106969** | **0.0189945** | **1.79E-08** | **0.0591** | **0.0075** | **3.01E-15** | **-0.0211** | **0.047** | **0.653301** |
| **rs1111875** | **-0.018979** | **0.018979** | **0.31731** | **-0.0948** | **0.0072** | **3.61E-39** | **-0.0092** | **0.0458** | **0.8402** |
| **rs11203203** | **0.149971** | **0.0189963** | **2.91E-15** | **0.0022** | **0.008** | **0.778999** | **0.0671** | **0.0509** | **0.1878** |
| **rs11708067** | **-0.0199986** | **0.0219985** | **0.363302** | **-0.0965** | **0.0086** | **5.93E-29** | **-0.0719** | **0.061** | **0.238** |
| **rs12416116** | **-0.164993** | **0.0209991** | **3.93E-15** | **0.0094** | **0.0087** | **0.2814** | **0.0064** | **0.0534** | **0.9049** |
| **rs12927355** | **-0.192008** | **0.0200009** | **7.99E-22** | **-0.0045** | **0.0083** | **0.5846** | **-0.03** | **0.0495** | **0.5446** |
| **rs12970134** | **0.0289761** | **0.0209827** | **0.167293** | **0.0555** | **0.008** | **5.31E-12** | **-0.0619** | **0.0578** | **0.2838** |
| **rs1456988** | **-0.111005** | **0.0200008** | **2.86E-08** | **0.0108** | **0.0087** | **0.2179** | **0.0127** | **0.0468** | **0.786901** |
| **rs1701704** | **0.222984** | **0.0189986** | **8.25E-32** | **-0.0111** | **0.0083** | **0.1823** | **-0.0756** | **0.0488** | **0.1216** |
| **rs17168486** | **0.019018** | **0.0250237** | **0.447255** | **0.0742** | **0.0094** | **2.18E-15** | **-0.1415** | **0.0569** | **0.0129101** |
| **rs17405722** | **0.00796817** | **0.0348607** | **0.819202** | **0.087** | **0.0146** | **2.28E-09** | **0.0605** | **0.0753** | **0.4216** |
| **rs1893217** | **0.192024** | **0.024003** | **1.24E-15** | **0.0185** | **0.0096** | **0.0531496** | **0.0858** | **0.0625** | **0.1698** |
| **rs2111485** | **0.164993** | **0.0189991** | **3.81E-18** | **-0.0056** | **0.0074** | **0.446** | **0.0782** | **0.0462** | **0.0905399** |
| **rs2237892** | **-0.0269602** | **0.037944** | **0.477378** | **-0.096** | **0.0157** | **8.75E-10** | **-0.145** | **0.1045** | **0.1654** |
| **rs229533** | **0.106969** | **0.0189945** | **1.79E-08** | **0.0039** | **0.0079** | **0.623799** | **0.07** | **0.0464** | **0.1316** |
| **rs2493394** | **0.138021** | **0.0290045** | **1.95E-06** | **0.073** | **0.0113** | **1.15E-10** | **0.0874** | **0.0661** | **0.1861** |
| **rs2611215** | **-0.167969** | **0.0249954** | **1.82E-11** | **0.0113** | **0.0107** | **0.291** | **-0.0866** | **0.0537** | **0.1064** |
| **rs2867125** | **0.0269602** | **0.0249631** | **0.280143** | **0.0601** | **0.0096** | **4.33E-10** | **0.0278** | **0.062** | **0.6536** |
| **rs3087243** | **-0.178051** | **0.0190054** | **7.36E-21** | **7.00E-04** | **0.0072** | **0.9243** | **-0.0691** | **0.0484** | **0.1532** |
| **rs3184504** | **-0.266051** | **0.0190036** | **1.56E-44** | **-0.024** | **0.0073** | **0.001008** | **-0.0885** | **0.0464** | **0.0564196** |
| **rs340874** | **-0.00697561** | **0.0189338** | **0.712559** | **0.0626** | **0.0073** | **8.41E-18** | **0.0227** | **0.0462** | **0.624001** |
| **rs3802177** | **0.0379699** | **0.0199842** | **0.0574328** | **-0.1217** | **0.008** | **2.32E-52** | **0.0061** | **0.047** | **0.8963** |
| **rs4820830** | **-0.134968** | **0.0189955** | **1.20E-12** | **-0.0072** | **0.0079** | **0.3632** | **-0.1354** | **0.0491** | **0.00583096** |
| **rs516246** | **0.142947** | **0.018993** | **5.22E-14** | **-0.0121** | **0.0074** | **0.1037** | **-0.0139** | **0.047** | **0.768** |
| **rs516946** | **-0.0230327** | **0.0210299** | **0.273412** | **0.0824** | **0.0085** | **3.16E-22** | **-0.0093** | **0.0562** | **0.8687** |
| **rs5215** | **0.0119714** | **0.0189547** | **0.527662** | **-0.0678** | **0.0073** | **2.09E-20** | **0.0377** | **0.0458** | **0.4095** |
| **rs56994090** | **-0.12897** | **0.0189956** | **1.13E-11** | **-0.0143** | **0.008** | **0.0720493** | **-0.0408** | **0.0458** | **0.3725** |
| **rs6043409** | **0.126017** | **0.0200027** | **2.98E-10** | **-0.0035** | **0.0082** | **0.670101** | **-0.0333** | **0.0497** | **0.5031** |
| **rs6059662** | **0.002002** | **0.019019** | **0.916167** | **0.0446** | **0.0079** | **1.51E-08** | **-0.0161** | **0.0546** | **0.768399** |
| **rs61839660** | **-0.471925** | **0.0359943** | **2.84E-39** | **-0.021** | **0.0132** | **0.1118** | **0.1293** | **0.1145** | **0.2585** |
| **rs62447205** | **-0.116983** | **0.020997** | **2.53E-08** | **0.0046** | **0.0088** | **0.5966** | **-0.0275** | **0.0489** | **0.5739** |
| **rs6679677** | **0.635995** | **0.0269998** | **1.10E-122** | **0.0517** | **0.0116** | **8.03E-06** | **0.0618** | **0.0648** | **0.3401** |
| **rs6691977** | **0.126016** | **0.0230029** | **4.30E-08** | **-0.0037** | **0.0098** | **0.7097** | **-0.0069** | **0.0487** | **0.8877** |
| **rs67232546** | **-0.0250102** | **0.0230094** | **0.277056** | **0.0596** | **0.0096** | **4.66E-10** | **-0.0026** | **0.0638** | **0.9678** |
| **rs6767484** | **-0.00803217** | **0.0200804** | **0.689157** | **0.1209** | **0.0076** | **2.70E-56** | **-0.021** | **0.0493** | **0.6693** |
| **rs6795735** | **-0.0179603** | **0.0189581** | **0.343451** | **-0.0558** | **0.0073** | **1.63E-14** | **0.1298** | **0.0471** | **0.005851** |
| **rs6827756** | **-0.131028** | **0.0190041** | **5.40E-12** | **-0.0113** | **0.0082** | **0.1677** | **0.0181** | **0.0456** | **0.6912** |
| **rs6960043** | **0.026036** | **0.0190263** | **0.17118** | **0.064** | **0.0071** | **3.61E-19** | **0.0048** | **0.0464** | **0.918** |
| **rs7144011** | **0.0280042** | **0.0220033** | **0.203115** | **0.0482** | **0.0085** | **1.64E-08** | **0.0791** | **0.0532** | **0.1365** |
| **rs7239671** | **0.120978** | **0.0179967** | **1.79E-11** | **0.0044** | **0.0078** | **0.5716** | **0.009** | **0.0459** | **0.8441** |
| **rs72727394** | **0.138021** | **0.0220034** | **3.55E-10** | **0.0431** | **0.0097** | **9.08E-06** | **-0.0784** | **0.0566** | **0.1658** |
| **rs72928038** | **0.179985** | **0.0239981** | **6.38E-14** | **0.0095** | **0.0102** | **0.3533** | **0.0145** | **0.0721** | **0.8412** |
| **rs7561798** | **0.0289761** | **0.0179852** | **0.107156** | **0.04** | **0.0072** | **2.79E-08** | **0.08** | **0.0457** | **0.07993** |
| **rs7607777** | **-0.0509776** | **0.0309864** | **0.0999355** | **-0.137** | **0.0125** | **9.40E-28** | **0.1884** | **0.1028** | **0.0669407** |
| **rs7729395** | **-0.0859937** | **0.0429969** | **0.0454999** | **0.1373** | **0.016** | **1.10E-17** | **-0.1522** | **0.0995** | **0.126** |
| **rs7756992** | **0.0240095** | **0.0210083** | **0.253098** | **0.1297** | **0.0078** | **6.00E-62** | **0.0061** | **0.0481** | **0.8995** |
| **rs780094** | **0.0360418** | **0.019022** | **0.0581273** | **0.0692** | **0.0074** | **5.16E-21** | **0.0106** | **0.0478** | **0.8243** |
| **rs7845219** | **-0.0280042** | **0.0190028** | **0.140567** | **-0.0422** | **0.0072** | **4.54E-09** | **0.0104** | **0.0455** | **0.8187** |
| **rs8056814** | **0.27801** | **0.0310012** | **3.03E-19** | **-0.113** | **0.0143** | **3.25E-15** | **-0.0095** | **0.0832** | **0.9088** |
| **rs825476** | **0.0160278** | **0.019033** | **0.399729** | **0.0524** | **0.0073** | **6.80E-13** | **0.0835** | **0.0465** | **0.0723802** |
| **rs849135** | **0.0619818** | **0.0189944** | **0.00110179** | **-0.0999** | **0.0072** | **1.04E-43** | **-0.0309** | **0.046** | **0.5019** |
| **rs853974** | **-0.0629748** | **0.0209916** | **0.00269979** | **-0.0601** | **0.0088** | **7.86E-12** | **0.0292** | **0.0512** | **0.5687** |
| **rs9585056** | **-0.116004** | **0.0210007** | **3.32E-08** | **0.0104** | **0.009** | **0.2495** | **0.0037** | **0.0524** | **0.9436** |
| **rs9928094** | **-0.00300451** | **0.0190286** | **0.87454** | **0.1045** | **0.0072** | **3.59E-47** | **0.0495** | **0.0459** | **0.2809** |
| **rs9940149** | **-0.002002** | **0.024024** | **0.933587** | **-0.058** | **0.0095** | **9.29E-10** | **-0.0346** | **0.0746** | **0.6428** |

**Supplementary Table 4.** STROBE-MR checklist of recommended items to address in reports of Mendelian randomization studies.

| **Item No.** | **Section** | **Checklist item** | **Page No.** | **Relevant text from manuscript** |
| --- | --- | --- | --- | --- |
| 1 | **TITLE and ABSTRACT** | Indicate Mendelian randomization (MR) as the study’s design in the title and/or the abstract if that is a main purpose of the study | 1 | Title: Diabetes mellitus and idiopathic pulmonary fibrosis: A Mendelian randomization study |
|  | **INTRODUCTION** |  |  |  |
| 2 | **Background** | Explain the scientific background and rationale for the reported study. What is the exposure? Is a potential causal relationship between exposure and outcome plausible? Justify why MR is a helpful method to address the study question | 4 | A meta-analysis of observational studies examining the relationship between diabetes mellitus (DM) and idiopathic pulmonary fibrosis (IPF) revealed that individuals with IPF had a 1.54-fold higher likelihood of developing DM than those without IPF; nevertheless, whether DM increases the risk of IPF remains controversial. Mendelian randomization (MR) studies have observational designs that employ randomly-assigned genetic variants as phenotypic instrumental variables (IVs) to establish reliable causal inferences regarding exposures and outcomes. Compared to traditional observational studies, MR is less susceptible to confounding or reverse causation. |
| 3 | **Objectives** | State specific objectives clearly, including pre-specified causal hypotheses (if any). State that MR is a method that, under specific assumptions, intends to estimate causal effects | 1 | This study aimed to investigate the causal association between type 1 diabetes (T1D), type 2 diabetes (T2D), and IPF using Mendelian randomization (MR) analysis. |
|  | **METHODS** |  |  |  |
| 4 | **Study design and data sources** | Present key elements of the study design early in the article. Consider including a table listing sources of data for all phases of the study. For each data source contributing to the analysis, describe the following: | 4, 5 | The causal relationships between T1D and IPF, as well as between T2D and IPF in the European population, were initially investigated using a two-sample univariate MR analysis. A sensitivity analysis was conducted to ensure data reliability and validity. Subsequently, MVMR analysis was conducted to examine the significance of the causal relationship between diabetes and IPF established by the univariate MR analysis. The detailed characteristics of the exposure and outcome datasets are presented in Table 1. |
|  | a) | Setting: Describe the study design and the underlying population, if possible. Describe the setting, locations, and relevant dates, including periods of recruitment, exposure, follow-up, and data collection, when available. | 5 | The T1D and T2D datasets were obtained from the European Bioinformatics Institute. The T1D dataset (ID number: "ebi-a-GCST005536") comprises 6,683 cases and 12,731 controls. The T2D dataset (ID number: "ebi-a-GCST006867") comprises 61,714 cases and 11,78 controls. The GWAS dataset associated with IPF was established by FinnGen Biobank (ID number: "finn-b-IPF"), and it comprises 1,028 cases and 196,986 controls . The detailed characteristics of the exposure and outcome datasets are presented in Table 1. |
|  | b) | Participants: Give the eligibility criteria, and the sources and methods of selection of participants. Report the sample size, and whether any power or sample size calculations were carried out prior to the main analysis | 5, 6 | The T1D dataset comprises 29,652 samples and 101,101 SNPs (https://gwas.mrcieu.ac.uk/datasets/ ebi-a-GCST005536/). The T2D datasetcomprises 655,666 samples and 5,030,727 SNPs (https://gwas.mrcieu.ac.uk/datasets/ ebi-a-GCST006867/). The GWAS dataset associated with IPF was established by FinnGen Biobank, and it comprises 198,014 samples and 16,380,413 SNPs (https://gwas.mrcieu.ac.uk/datasets/finn-b-IPF/). The diagnosis of IPF and DM was determined according to the International Classification of Diseases 10th Revision (ICD-10）codes. |
|  | c) | Describe measurement, quality control and selection of genetic variants | 6 | A genome-wide search was conducted for SNPs (P<5×10^-8^) associated with T1D or T2D exposure, which were used as IVs for genetic prediction. Non-independent SNPs based on linkage disequilibrium (r^2^<0.001 within a 10,000 kb aggregation window) were excluded using the European population reference. To ensure independence and exclusivity, these SNPs were searched using Phenoscanner (University of Cambridge, Cambridge, UK; http://www.phenoscanner.medschl.cam.ac.uk/) and any SNPs related to the outcome or confounding factors were excluded. |
|  | d) | For each exposure, outcome, and other relevant variables, describe methods of assessment and diagnostic criteria for diseases | 5 | The diagnosis of IPF and DM was determined according to the International Classification of Diseases 10th Revision (ICD-10）codes. |
|  | e) | Provide details of ethics committee approval and participant informed consent, if relevant | 8 | This study relied solely on published GWAS datasets, and all original studies obtained appropriate ethical approval; therefore, separate ethical approval was not required for this study. |
| 5 | **Assumptions** | Explicitly state the three core IV assumptions for the main analysis (relevance, independence and exclusion restriction) as well assumptions for any additional or sensitivity analysis | 5 | We applied a set of selection criteria to identify single nucleotide polymorphisms (SNPs) strongly associated with T1D and T2D, which were subsequently used as IVs in the MR analysis: (I) strong association between IVs and the exposure of interest; (II) no direct relationship between IVs and outcome, with their effect only indicated through exposure; and (III) independence of IVs from any confounding factors affecting both exposure and outcome. |
| 6 | **Statistical methods: main analysis** | Describe statistical methods and statistics used | 7, 8 | In the univariate MR analysis, we employed inverse variance-weighted (IVW), weighted median (WM), and MR-Egger regression methods. For the MVMR analysis, we used the multivariate IVW method primarily, and supplemented it with multivariate MR-Egger and multivariate MR- least absolute shrinkage and selection operator methods. Heterogeneity tests were conducted using the MR-IVW and MR-Egger regression methods, whereas pleiotropic effects were assessed using the MR-Egger intercept. |
|  | a) | Describe how quantitative variables were handled in the analyses (i.e., scale, units, model) |  | Statistical effect sizes or units of measurement for exposures, outcomes, and relevant covariates were not transformed and are not reported |
|  | b) | Describe how genetic variants were handled in the analyses and, if applicable, how their weights were selected | 6 | Based on GWAS in online databases, specific methods for screening instrumental variables were described in this article. |
|  | c) | Describe the MR estimator (e.g. two-stage least squares, Wald ratio) and related statistics. Detail the included covariates and, in case of two-sample MR, whether the same covariate set was used for adjustment in the two samples | 7, 8 | In this paper, the MR Statistical methods and the software used are described in detail. |
|  | d) | Explain how missing data were addressed |  | Not applicable. |
|  | e) | If applicable, indicate how multiple testing was addressed | 8 | MVMR analysis was conducted to examine the significance of the causal relationship between diabetes and IPF established by the univariate MR analysis. |
| 7 | **Assessment of assumptions** | Describe any methods or prior knowledge used to assess the assumptions or justify their validity | 6 | To ensure a strong relationship between the IVs and exposure, it is necessary to calculate F-statistics and R2. |
| 8 | **Sensitivity analyses and additional analyses** | Describe any sensitivity analyses or additional analyses performed (e.g. comparison of effect estimates from different approaches, independent replication, bias analytic techniques, validation of instruments, simulations) | 7 | To ensure the reliability of our research findings, we employed MR-IVW and MR-Egger regression methods to assess heterogeneity and quantified the results using Cochran's Q statistics in univariable MR. In MVMR, the extended IVW and MR-Egger methods were employed to assess heterogeneity, while the MR-Egger intercept was used to evaluate pleiotropic effects. |
| 9 | **Software and pre-registration** |  |  |  |
|  | a) | Name statistical software and package(s), including version and settings used | 8 | The statistical analyses in this study were performed using the "TwoSampleMR" and "MendelianRandomization" packages in R v4.3.0 (R Foundation for Statistical Computing, Vienna, Austria). |
|  | b) | State whether the study protocol and details were pre-registered (as well as when and where) |  | No preregistration was required for the study protocol and details |
|  | **RESULTS** |  |  |  |
| 10 | **Descriptive data** |  |  |  |
|  | a) | Report the numbers of individuals at each stage of included studies and reasons for exclusion. Consider use of a flow diagram | 24 | Demographic data such as the number of cases in each data set included in the study are shown in Table 1. |
|  | b) | Report summary statistics for phenotypic exposure(s), outcome(s), and other relevant variables (e.g. means, SDs, proportions) |  | Relevant summary statistics were not available |
|  | c) | If the data sources include meta-analyses of previous studies, provide the assessments of heterogeneity across these studies |  | Data sources did not include meta-analyses of previous studies |
|  | d) | For two-sample MR:  i.  Provide justification of the similarity of the genetic variant-exposure associations between the exposure and outcome samples  ii.  Provide information on the number of individuals who overlap between the exposure and outcome studies | 5, 24 | i.  All the sample populations listed in Table 1 were from Europe and thus showed little ethnic heterogeneity  ii.  We utilized GWAS datasets from various databases for exposure and outcome to mitigate potential bias in causal effect estimation due to sample overlap. |
| 11 | **Main results** |  |  |  |
|  | a) | Report the associations between genetic variant and exposure, and between genetic variant and outcome, preferably on an interpretable scale |  | Relationships among exposures, outcomes, and covariates were interpreted in directed acyclic graphs( Figure 1). |
|  | b) | Report MR estimates of the relationship between exposure and outcome, and the measures of uncertainty from the MR analysis, on an interpretable scale, such as odds ratio or relative risk per SD difference | 8, 9 | In the two-sample univariate MR analysis, the IVW analysis revealed that T1D was associated with an increased risk of IPF (OR = 1.118, 95% CI = 1.021–1.225, P = 0.016), with a mean increase of 11.8% of the likelihood of developing IPF. When T1D was adjusted for T2D, the results of the multivariate analysis were consistent with the results of the univariate MR, suggesting that T1D was significantly associated with an increased risk of IPF (IVW: OR = 1.133, 95% CI = 1.011–1.270, P = 0.032, MR-Lasso: OR = 1.114, 95% CI = 1.004–1.236, P = 0.042, Table 3 and figure 6). |
|  | c) | If relevant, consider translating estimates of relative risk into absolute risk for a meaningful time period |  | Not applicable. |
|  | d) | Consider plots to visualize results (e.g. forest plot, scatterplot of associations between genetic variants and outcome versus between genetic variants and exposure) |  | Visualizations of the results are shown in Figure 2-6(including forest plots, etc.) |
| 12 | **Assessment of assumptions** |  |  |  |
|  | a) | Report the assessment of the validity of the assumptions | 6 | A genome-wide search was conducted for SNPs (P<5×10-8) associated with T1D or T2D exposure, which were used as IVs for genetic prediction. Non-independent SNPs based on linkage disequilibrium (r2<0.001 within a 10,000 kb aggregation window) were excluded using the European population reference. To ensure independence and exclusivity, these SNPs were searched using Phenoscanner and any SNPs related to the outcome or confounding factors were excluded. Confounding factors included risk factors other than exposure that may contribute to the outcome. The risk factors associated with IPF include smoking, gastroesophageal reflux, obstructive sleep apnea, herpes virus infection, and certain occupational interstitial lung diseases. To ensure a strong relationship between the IVs and exposure, it is necessary to calculate F-statistics and R2. |
|  | b) | Report any additional statistics (e.g., assessments of heterogeneity across genetic variants, such as I2, Q statistic or E-value) | 24 | Details of the results of the assessment of heterogeneity of genetic variants are provided in Table 4 |
| 13 | **Sensitivity analyses and additional analyses** |  |  |  |
|  | a) | Report any sensitivity analyses to assess the robustness of the main results to violations of the assumptions | 7, 8 | we employed MR-IVW and MR-Egger regression methods to assess heterogeneity and quantified the results using Cochran's Q statistics. We employed the MR-Egger intercept test to examine the presence of horizontal pleiotropy while utilizing a scatter plot of effective IVs for estimation of the causal effect of T1D and T2D on IPF to visually assess whether the outcome effect is zero when the IV effect is zero. In MVMR analysis, the extended IVW and MR-Egger methods were employed to assess heterogeneity, while the MR-Egger intercept was used to evaluate pleiotropic effects. |
|  | b) | Report results from other sensitivity analyses or additional analyses | 10 | The effective IVs used to estimate the effect of T1D on IPF showed no heterogeneity (IVW: I^2^ =0.14, P=0.232; MR-Egger: I^2^ =0.15, P=0.217, Table 4). The effective IVs used to estimate the effect of T2D on IPF also showed no heterogeneity (I^2^ = 0, P > 0.05, Table 4). The MR-Egger intercepts for the effects of T1D and T2D on IPF were close to 0, with P-values of 0.464 and 0.777, respectively, suggesting no substantial horizontal pleiotropic bias (Figure 2). In addition, the leave-one-out analysis showed that no single SNP in T1D or T2D on IPF affected causal effect estimates (Figures 3 and 4), whereas effect SNPs in the univariate MR analysis were approximately symmetrical in a funnel plot (Figure 2). The results of the MVMR heterogeneity test showed no significant heterogeneity and no pleiotropic bias (MR-IVW: P = 0.125, MR-Egger: P = 0.109; MR-Egger intercept estimate = 0.003, P = 0.758). |
|  | c) | Report any assessment of direction of causal relationship (e.g., bidirectional MR) | 17, 18 | We examined the causal impact of both diabetes types on IPF without exploring the reverse causal effect. When we performed a reverse causality analysis, only six SNPs were screened from the IPF dataset, and these six SNPs did not correspond with the GWAS of T1D and T2D. |
|  | d) | When relevant, report and compare with estimates from non-MR analyses |  | Non-MR analyses were not performed in this article. |
|  | e) | Consider additional plots to visualize results (e.g., leave-one-out analyses) |  | Figure 2 to Figure 6 are the visualization results (including scatter plot, funnel plot, forest plot, leave-one-out analyses). |
|  | **DISCUSSION** |  |  |  |
| 14 | **Key results** | Summarize key results with reference to study objectives | 10 | In this study, we assessed the causal effects of T1D and T2D on IPF using a two-sample univariate MR approach. We identified a causal relationship between T1D and IPF, but no such association was observed between T2D and IPF. Furthermore, the findings of the MVMR analysis were consistent with those of the univariate analysis: namely, that only T1D was associated with an increased risk of IPF. |
| 15 | **Limitations** | Discuss limitations of the study, taking into account the validity of the IV assumptions, other sources of potential bias, and imprecision. Discuss both direction and magnitude of any potential bias and any efforts to address them | 17, 18 | First, we examined the causal impact of both diabetes types on IPF without exploring the reverse causal effect. When we performed a reverse causality analysis, only six SNPs were screened from the IPF dataset, and these six SNPs did not correspond with the GWAS of T1D and T2D. However, our causal inferences about the two types of diabetes and IPF were reliable; the F-statistics of the IVs we selected were all greater than 10, and the sensitivity analysis did not suggest the existence of significant heterogeneity or pleiotropy. |
| 16 | **Interpretation** |  |  |  |
|  | a) | Meaning: Give a cautious overall interpretation of results in the context of their limitations and in comparison with other studies | 10 | We identified a causal relationship between T1D and IPF, but no such association was observed between T2D and IPF. Furthermore, the findings of the MVMR analysis were consistent with those of the univariate analysis: namely, that only T1D was associated with an increased risk of IPF. |
|  | b) | Mechanism: Discuss underlying biological mechanisms that could drive a potential causal relationship between the investigated exposure and the outcome, and whether the gene-environment equivalence assumption is reasonable. Use causal language carefully, clarifying that IV estimates may provide causal effects only under certain assumptions | 13, 15 | The possible roles of KAT2B, citrullination, and hyperglycemia in the potential causal relationship between exposure and outcome are described. |
|  | c) | Clinical relevance: Discuss whether the results have clinical or public policy relevance, and to what extent they inform effect sizes of possible interventions | 11, 12 | Our univariate and multivariate MR analyses indicated that T1D is associated with an average increase in IPF risk of 11.8% and 13.3%, respectively, and genetically-predicted T1D increases the risk of developing IPF. And our study revealed no causal relationship between T2D and IPF; however, the results of previous observational studies on the association between these two conditions have been controversial. We also discuss the association between diabetes and IPF in clinical studies. |
| 17 | **Generalizability** | Discuss the generalizability of the study results (a) to other populations, (b) across other exposure periods/timings, and (c) across other levels of exposure | 18 | Furthermore, owing to the utilization of GWAS summary-level data instead of individual-level data, we were unable to investigate the impact of variables such as sex, age, and specific types of exposure on outcome or stratify the impact of different glycemic control conditions on IPF. The outcome population in our study consisted of the FinnGen population, although of European origin, represents a distinct and extant group, which may limit the generalization of our conclusions. |
|  | **OTHER INFORMATION** |  |  |  |
| 18 | **Funding** | Describe sources of funding and the role of funders in the present study and, if applicable, sources of funding for the databases and original study or studies on which the present study is based | 20 | We did not receive any funding for this study. |
| 19 | **Data and data sharing** | Provide the data used to perform all analyses or report where and how the data can be accessed, and reference these sources in the article. Provide the statistical code needed to reproduce the results in the article, or report whether the code is publicly accessible and if so, where | 20 | The GWAS summary datasets for T1D (GWAS ID: ebi-a-GCST005536), T2D (GWAS ID: ebi-a-GCST006867), and IPF (GWAS ID: finn-b-IPF) are available through the ieu open gwas project (https://gwas.mrcieu.ac.uk/datasets). |
| 20 | **Conflicts of Interest** | All authors should declare all potential conflicts of interest | 20 | The authors declare that they have no competing interests |

This checklist is copyrighted by the Equator Network under the Creative Commons Attribution 3.0 Unported (CC BY 3.0) license.

1. Skrivankova VW, Richmond RC, Woolf BAR, Yarmolinsky J, Davies NM, Swanson SA, et al. Strengthening the Reporting of Observational Studies in Epidemiology using Mendelian Randomization (STROBE-MR) Statement. JAMA. 2021;under review.

2. Skrivankova VW, Richmond RC, Woolf BAR, Davies NM, Swanson SA, VanderWeele TJ, et al. Strengthening the Reporting of Observational Studies in Epidemiology using Mendelian Randomisation (STROBE-MR): Explanation and Elaboration. BMJ. 2021;375:n2233.

**Supplementary File.** The main code used in the MR analyses.

library("devtools")

library(TwoSampleMR)

t1d_exp_dat <- extract_instruments(outcomes='ebi-a-GCST005536')

t1d_exp_dat<-extract_instruments(outcomes='ebi-a-GCST005536',

clump=TRUE, r2=0.001,

kb=10000,access_token = NULL, p1=5e-8)

ipf_out_dat <- extract_outcome_data(snps = t1d_exp_dat$SNP, outcomes = 'finn-b-IPF')

dat <- harmonise_data(t1d_exp_dat, ipf_out_dat)

dat$se.outcome=sqrt(((dat$beta.outcome)^2)/qchisq(dat$pval.outcome,1,lower.tail=F))

mr(dat)

res <- mr(dat)

generate_odds_ratios(res)

mr_heterogeneity(dat)

mr_heterogeneity(dat, method_list=c("mr_egger_regression", "mr_ivw"))

pleio<-mr_pleiotropy_test(dat)

res_single <- mr_singlesnp(dat)

res_loo <- mr_leaveoneout(dat)

head(res)

generate_odds_ratios(res)

res<-mr(dat)

write.csv(dat,"t1ddata.csv")

write.csv(res,"t1dresult.csv")

res <- mr(dat)

p1 <- mr_scatter_plot(res, dat)

p1[[1]]

length(p1)

ggsave(p1[[1]], file="t1dscatter.png", width=7, height=7)

res_single <- mr_singlesnp(dat)

p2 <- mr_forest_plot(res_single)

p2[[1]]

ggsave(p2[[1]], file="t1dforest.png", width=7, height=7)

res_loo <- mr_leaveoneout(dat)

p3 <- mr_leaveoneout_plot(res_loo)

p3[[1]]

ggsave(p3[[1]], file="t1dleaveoneout.png", width=7, height=7)

res_single <- mr_singlesnp(dat)

p4 <- mr_funnel_plot(res_single)

p4[[1]]

ggsave(p4[[1]], file="t1dfunnel.png", width=7, height=7)

library("devtools")

library(TwoSampleMR)

t2d_exp_dat <- extract_instruments(outcomes=' ebi-a-GCST006867')

t2d_exp_dat<-extract_instruments(outcomes=' ebi-a-GCST006867',

clump=TRUE, r2=0.001,

kb=10000,access_token = NULL, p1=5e-8)

ipf_out_dat <- extract_outcome_data(snps = t2d_exp_dat$SNP, outcomes = 'finn-b-IPF')

data <- harmonise_data(t2d_exp_dat, ipf_out_dat)

data$se.outcome=sqrt(((data$beta.outcome)^2)/qchisq(data$pval.outcome,1,lower.tail=F))

mr(data)

res2 <- mr(data)

generate_odds_ratios(res2)

mr_heterogeneity(data)

mr_heterogeneity(data, method_list=c("mr_egger_regression", "mr_ivw"))

pleio<-mr_pleiotropy_test(data)

res2_single <- mr_singlesnp(data)

res2_loo <- mr_leaveoneout(data)

head(res2)

generate_odds_ratios(res2)

res2<-mr(data)

write.csv(dat,"t2ddata.csv")

write.csv(res," t2dresult.csv")

res2 <- mr(data)

p1 <- mr_scatter_plot(res2, data)

p1[[1]]

length(p1)

ggsave(p1[[1]], file="t2dscatter.png", width=7, height=7)

res2_single <- mr_singlesnp(dat)

p2 <- mr_forest_plot(res_single)

p2[[1]]

ggsave(p2[[1]], file="t2dforest.png", width=7, height=7)

res_loo <- mr_leaveoneout(dat)

p3 <- mr_leaveoneout_plot(res_loo)

p3[[1]]

ggsave(p3[[1]], file="t2dleaveoneout.png", width=7, height=7)

res_single <- mr_singlesnp(dat)

p4 <- mr_funnel_plot(res_single)

p4[[1]]

ggsave(p4[[1]], file="t2dfunnel.png", width=7, height=7)

library(MendelianRandomization)

library(MVMR)

library(TwoSampleMR)

id_exposure <- c("ebi-a-GCST005536","ebi-a-GCST006867")

id_outcome <- "finn-b-IPF"

exposure_dat <- mv_extract_exposures(id_exposure)

outcome_dat <- extract_outcome_data(exposure_dat$SNP, id_outcome)

MRMVInputObject <- mr_mvinput(bx = exposure_dat$Beta,

bxse = exposure_ dat $SE,

by = outcome_dat $Beta,

byse = outcome_dat $SE)

MRMVInputObject

MRMVObject <- mr_mvivw(MRMVInputObject,

model = "default",

correl = FALSE,

distribution = "normal",

alpha = 0.05)

MRMVObject<-mr_mvegger(

MRMVInputObject,

orientate = 1,

correl = FALSE,

distribution = "normal",

alpha = 0.05)

MRMVObject<-mr_mvlasso(

MRMVInputObject,

orientate = 1,

distribution = "normal",

alpha = 0.05,

lambda = numeric(0)

)

MRMVObject<-mr_mvmedian(

MRMVInputObject,

distribution = "normal",

alpha = 0.05,

iterations = 10000,

seed = 314159265

)
